# Tumor-derived WNT7A reprograms pulmonary fibroblasts to remodel the metastatic niche and promote bladder cancer lung metastasis

**DOI:** 10.1038/s12276-026-01735-x

**Published:** 2026-06-03

**Authors:** Zhengnan Huang, Yilin Yan, Xinan Wang, Huaxing Li, Jingming Zhuang, Xiangqian Cao, Yang Wang, Denglong Wu, Bing Shen

**Affiliations:** 1 Department of Urology, Tongji Hospital, School of Medicine, Tongji University, Shanghai, China; 2 Department of Urology, Shanghai Ninth People’s Hospital, Shanghai Jiaotong University School of Medicine, Shanghai, China; 3 Department of Urology, Shanghai Tenth People’s Hospital, School of Medicine, Tongji University, Shanghai, China; 4 Department of Urology, Shanghai General Hospital, Shanghai Jiaotong University School of Medicine, Shanghai, China; 5 Urologic Cancer Institute, School of Medicine, Tongji University, Shanghai, China; 6 Tongji University Cancer Center, Shanghai Tenth People’s Hospital, School of Medicine, Tongji University, Shanghai, China

**Keywords:** Bladder cancer, Cancer microenvironment

## Abstract

The mechanisms underlying lung metastases in bladder cancer (BLCA) remain poorly understood. Cancer-associated fibroblasts (CAFs) are key modulators of the metastatic microenvironment, but how they are activated and contribute to BLCA lung metastases remains unclear. Single-nucleus RNA sequencing was conducted on metastatic lung lesions from patients with BLCA to characterize the tumor microenvironment. Functional and molecular biology experiments, including co-culture assays, luciferase reporter assays, chromatin immunoprecipitation and in vivo lung metastasis models, were performed to explore the mechanisms by which tumor-derived factors and CAFs-secreted exosomes contribute to metastasis. The results revealed that CAFs were enriched in metastatic lung lesions and activated by tumor-derived WNT7A via the Wnt/β-catenin pathway. These activated CAFs promoted BLCA cell proliferation, stemness and migration through the exosomal delivery of miR-1910-5p, which directly suppressed CTDNEP1 expression and activated MYC signaling. Mechanistically, RBMX was identified as a regulator of miR-1910-5p packaging into CAFs-derived exosomes. In vivo, inhibition of CAFs-derived exosomes secretion reduced lung metastasis, highlighting their critical role in metastasis formation. In conclusion, this study uncovers a novel reciprocal activation loop between tumor cells and CAFs in BLCA lung metastases, where tumor-secreted WNT7A activates resident pulmonary CAFs, which in turn enhance tumor malignancy through exosomal delivery of miR-1910-5p. This exosome-mediated crosstalk promotes metastatic progression via the CTDNEP1/MYC signaling pathway. These findings provide potential therapeutic targets for mitigating metastatic progression in BLCA.

## Tumor Factors Activate CAFs to Drive Bladder Cancer Metastasis

Bladder cancer frequently metastasizes to the lungs, a process driven by complex interactions within the tumor microenvironment. This study uncovers the role of tumor-derived WNT7A in transforming normal fibroblasts into cancer-associated fibroblasts (CAFs), which remodel the metastatic niche. Researchers used single-nucleus RNA sequencing and various in vitro assays to demonstrate that WNT7A activates the Wnt/β-catenin signaling pathway, upregulating markers such as α-SMA and FAP in fibroblasts. This activation enhances fibroblast contractility and supports bladder cancer cell colonization in the lungs. Notably, CAFs release exosomes containing miR-1910-5p, which are transferred to bladder cancer cells, promoting malignancy by targeting CTDNEP1 and activating MYC signaling. These findings highlight a feedback loop where WNT7A-induced CAFs enhance bladder cancer aggressiveness, suggesting potential therapeutic targets for preventing lung metastasis. Future research could explore the broader implications of this signaling axis in other cancer types.

This summary was initially drafted using artificial intelligence, then revised and fact-checked by the author.

## Introduction

Lung metastasis is the most common event of distant metastases in bladder cancer (BLCA) and a leading cause of BLCA-related mortality^1,2^. Tumor metastasis is a complex, multi-stage process, with the colonization and subsequent proliferation in distant organs to form micrometastases being the most intricate and rate-limiting step^3^. Cancer stem-like cells from the primary tumor site, upon invading distant organs, can exploit and remodel the surrounding microenvironment. This remodeling enables the microenvironment to provide signals that sustain their proliferation and stemness, thus facilitating the colonization of cancer stem-like cells in distant organs^4^. Once remodeled to support tumor cell colonization, this microenvironment is referred to as the metastatic niche. The formation of the metastatic niche is a complex process involving multiple cellular interactions, in which fibroblasts, as a core component of this microenvironment, play a crucial role in its establishment. Normal fibroblasts (NFs), when stimulated by the tumor microenvironment, are activated and transdifferentiated into cancer-associated fibroblasts (CAFs). Activated CAFs regulate extracellular matrix remodeling by secreting various cytokines, chemokines, matrix metalloproteinases and other factors, thereby altering the physical and chemical properties of microenvironment and providing a favorable environment for tumor cell colonization^5^. The molecular mechanisms by which CAFs promote tumor metastasis have been elucidated. For instance, studies have shown that activated CAFs play a critical role in enabling the colonization of salivary adenoid cystic carcinoma cells in the lung^6^. Moreover, research has demonstrated that hepatocellular carcinoma cells can induce the activation and conversion of fibroblasts into CAFs, which subsequently secrete chemokines such as IL-6 to facilitate the lung metastasis of liver cancer.^7^

Exosomes, which are small membranous vesicles with a size range of 30–150 nm, serve as essential cellular communicators, encapsulating a diverse array of proteins, lipids, mRNAs, miRNAs and lncRNAs^8–10^. Extensive research has demonstrated that exosomes regulate the tumor microenvironment, thereby facilitating cancer metastasis and progression^11,12^. Among the various molecules carried by exosomes, miRNAs have garnered substantial attention due to their involvement in immunomodulation, chemoresistance and metastasis across multiple tumor types. miRNAs within exosomes can be internalized by adjacent or distant cells, where they promote oncogenic signaling by suppressing target mRNAs in the recipient cells^13,14^.

WNT7A, a member of the Wnt family, is a secreted glycoprotein that serves as a key component of the Wnt signaling pathway, playing pivotal roles in diverse physiological and pathological processes through pathway activation^15,16^. Our previous study revealed that WNT7A expression was significantly upregulated in BLCA tissues compared with normal bladder epithelium. Furthermore, WNT7A was shown to enhance the invasive capacity of BLCA cells by activating the Wnt signaling pathway. In vivo experiments further revealed that overexpression of WNT7A markedly promotes the lung metastatic potential of BLCA cells^17^. However, the molecular mechanisms underlying the role of WNT7A in facilitating lung metastasis in BLCA remain to be elucidated.

In this study, we aim to elucidate the molecular mechanisms underlying lung metastasis in BLCA. Through comprehensive analyses, we demonstrate that tumor-derived WNT7A plays a critical role in remodeling the metastatic microenvironment by inducing the activation of NFs into CAFs. miRNA microarray profiling identified miR-1910-5p as a key differentially expressed molecule in exosomes derived from NFs and CAFs. Subsequent functional studies revealed that exosomal miR-1910-5p is transferred from CAFs to BLCA cells, promoting malignant phenotypes and facilitating their colonization and metastatic outgrowth in the lung. These findings provide novel insights into the mechanisms driving BLCA lung metastasis and highlight a positive feedback loop wherein tumor-derived WNT7A reprograms fibroblasts to create a metastatic niche that, in turn, reinforces tumor cell aggressiveness.

## Materials and methods

### Patients and samples

All tissue samples utilized in this study were obtained from the Shanghai General Hospital. Informed consent was obtained from all participating patients before sample collection. The study protocol was reviewed and approved by the Ethical Committee of the Shanghai General Hospital.

### snRNA-seq

Lung metastatic tissue samples from patients with BLCA were collected, formalin-fixed, paraffin-embedded and stored for further processing. Nuclei isolation and permeabilization were carried out following the guidelines outlined in the Chromium Next Gel Beads-in-Emulsion (GEM) Single-Cell Multiome ATAC + Gene Expression User Guide (CG000338). Single-nucleus RNA sequencing (snRNA-seq) libraries were generated using the Chromium Single Cell 3ʹ Reagent Kits v3 (10× Genomics), adhering to the manufacturer’s protocol. The workflow included key steps such as nuclei counting and quality assessment, generation and barcoding of GEMs, post-GEM reverse transcription cleanup, cDNA amplification, construction of gene expression libraries and high-throughput sequencing on the NovaSeq platform (Illumina). In parallel, seven control lung tissue samples from GSE171524 dataset were included for comparative analysis with the metastatic lung lesions^18^.

### Cell culture

The human BLCA cell lines (T24, J82, 5637, RT4, UMUC-3, SCaBER and TCC-SUP) and the mouse BLCA cell line MB49 were obtained from the Cell Bank of the Chinese Academy of Sciences. T24, 5637 and MB49 cells were grown in RPMI-1640 medium (Gibco), RT4 cells were maintained in McCoy’s 5a Medium Modified (Gibco). J82, UMUC-3, SCaBER and TCC-SUP cells were cultured in Eagle’s minimum essential medium (Gibco). The fibroblast cell line MRC5, sourced from ScienCell Research Laboratories, was also grown in Eagle’s minimum Essential Medium (Gibco). All culture media were supplemented with 10% fetal bovine serum (FBS) and penicillin/streptomycin. The cells were incubated in a humidified atmosphere containing 5% CO_2_ at 37 °C.

### RNA interference and plasmids

Short-hairpin RNAs, small interfering RNAs (siRNAs), miR-1910-5p mimic and miR-1910-5p inhibitor, as well as WNT7A, MYC and CTDNEP1 constructs, were synthesized by GenePharma and subsequently cloned into lentiviral vectors or plasmids. Stable cell lines were established following lentiviral transduction and selection with 4 μg ml^−1^ puromycin. Cell transfections were performed using Lipofectamine 3000 (Invitrogen) as the transfection reagent, following the manufacturer’s protocol. Detailed sequences of the short-hairpin RNAs, siRNAs and miRNA mimic and inhibitor are listed in Supplementary Table 1.

### RNA extraction and real-time qPCR

Total RNA was extracted using TRIzol reagent (Invitrogen) and subsequently reverse-transcribed into cDNA with a reverse transcriptase kit (Vazyme). miRNA cDNA was synthesized from the total RNA using the miRcute Plus miRNA First-Strand cDNA Synthesis Kit (TIANGEN). Before extraction, the exogenous reference cel-miR-39 (TIANGEN) was added to the culture medium or exosomes. miRNA from these samples was then extracted using the mirVana PARIS Kit (Ambion). Quantitative PCR (qPCR) for mRNA was conducted using the ChamQ Universal SYBR qPCR Master Mix (Vazyme), whereas qPCR for miRNA was performed using the miRcute Plus miRNA qPCR Detection Kit (TIANGEN). In cell lysates, mRNA levels were normalized to β-actin and miRNA levels were normalized to U6. In culture medium and exosomes, miRNA levels were normalized to the exogenous reference cel-miR-39. For primers sequences used for qPCR, refer to Supplementary Table 1.

### Western blot

For cellular samples, cells were lysed in RIPA buffer (Beyotime) supplemented with phosphatase and protease inhibitors. For exosomal samples, BeyoExo Exosome Lysis Buffer (Beyotime) was added to the purified exosome suspension at the recommended ratio. Equal amounts of protein (20 μg) were equally loaded, separated via SDS–polyacrylamide gel electrophoresis and transferred to a PVDF membrane (Millipore). Following a blocking step with 5% non-fat milk, the membranes were incubated with the primary antibody overnight at 4°C. Subsequently, the membranes were treated with secondary antibodies, and the signals were detected using an Odyssey Infrared Imaging System (Biosciences). For details on the antibodies used, refer to Supplementary Table 2.

### ELISA

The concentration of WNT7A in the conditioned medium (SM) was measured using a WNT7A ELISA Kit (FineTest) following the manufacturer’s protocol. In brief, supernatants were centrifuged at 2,500 rpm for 5 min at 4 °C to remove debris. Samples and serially diluted standards were added to the ELISA plate and incubated at 37 °C for 90 min. After washing, biotin-conjugated antibody and HRP-streptavidin were sequentially added, each followed by incubation and washing steps. 3,3’,5,5’-Tetramethylbenzidine (TMB) substrate was added for color development, and the reaction was stopped with stop solution. Absorbance at 450 nm was measured, and WNT7A concentrations were calculated using a standard curve.

### Collagen contraction assay

Collagen gels were prepared in 24-well plates using a neutralization solution (Advanced BioMatrix) and rat-tail collagen type I (Advanced BioMatrix) according to the manufacturer’s protocol. Subsequently, fibroblasts were embedded within the collagen gels and incubated at 37 °C for 1 h to facilitate gel formation. Upon completion of polymerization, the collagen gels were carefully detached from the wells using a pipette tip and maintained in continuous incubation at 37 °C. The gels were photographed at specified intervals, and their diameters were measured to assess the extent of contraction.

### Colony formation assay

A total of 1 × 10^3^ cells were plated into six-well plates. Following a 14-day incubation period, the cells were fixed with formaldehyde and subsequently stained with crystal violet. The number of resulting colonies was then quantified.

### Wound-healing assay

Cells were seeded into six-well plates, after which the cell monolayers were wounded with a pipette tip to create a uniform gap. Images were captured at designated time points to assess the rate of wound closure.

### Migration and Invasion assay

Cell migration and invasion assays were performed using 8-μm pore-size transwell inserts chambers (Corning). Cells suspended in a serum-free medium were added to the upper chamber. For invasion assays, the upper chamber was coated with matrigel mix, while it remained uncoated for migration assays. The lower chamber was filled with culture medium containing 20% FBS. After a designated incubation period, cells that had invaded or migrated to the membrane’s underside were fixed, stained, and counted.

### Sphere formation assay

A total of 2 × 10^3^ cells were resuspended in ultra-low attachment plates (Corning) in serum-free DMEM/F12 medium supplemented with 20 μl/ml B27 (Gibco), 10 ng/ml basic fibroblast growth factor (PeproTech) and 10 ng/ml human recombinant epidermal growth factor (PeproTech). After a culture period of 7–10 days, the number and size of the spheres were examined under microscope, and representative images were taken. Colonies with diameters exceeding 50 μm were recorded as single-positive colonies.

### Immunofluorescence

Cells cultured on cover slips were fixed with 4% paraformaldehyde, permeabilized with 0.1% Triton X-100 and blocked with 3% BSA. Subsequently, the cells were incubated with primary antibodies overnight at 4 °C. The samples were then incubated with AF488- or AF647-conjugated anti-rabbit or anti-mouse IgG (Invitrogen) for 1 h at room temperature in the dark. Following this, nuclear counterstaining was performed using DAPI (Invitrogen). For details on the antibodies used, refer to Supplementary Table 2.

### IHC

Tissues were fixed in 4% paraformaldehyde, paraffin-embedded and sectioned. After antigen retrieval with citrate buffer and blocking with 5% BSA, sections were incubated overnight at 4 °C with primary antibodies, followed by secondary antibodies for 1 h at room temperature. 3,3′-diaminobenzidine (DAB) was used for visualization, and hematoxylin for counterstaining. Sections were dehydrated, cleared, mounted, imaged and evaluated. The detailed scoring criteria were as follows: each slide was assessed according to staining intensity (0, no staining; 1, weak; 2, moderate; 3, strong) and the percentage of positive tumor cells (1, 0–25%; 2, 26–50%; 3, 51–75%; 4, 76–100%). The final immunohistochemistry (IHC) score was calculated by multiplying the intensity score by the percentage score. Two certified pathologists independently evaluated each sample in a blinded manner, and the average of their scores was taken as the final result. For details on the antibodies used, refer to Supplementary Table 2.

### FISH

A fluorescence in situ hybridization Kit (RiboBio) was employed in line with the provided directions. In brief, tissue sections were deparaffinized with xylene and rehydrated through graded ethanol solutions. Antigen retrieval was performed by boiling the sections in sodium citrate buffer for 10 min. The sections were then permeabilized with 0.5% Triton X-100 at 4 °C for 15 min and washed three times with PBS. Subsequently, the sections were pre-hybridized at 37 °C in an RNA-Fluorescence In Situ Hybridization (FISH) Hybridization System (Novobiotec) for 30 min, followed by overnight incubation with a specific anti-miR-1910-5p oligodeoxynucleotide probe at 37 °C in the dark. After hybridization, the sections were washed with saline-sodium citrate buffer and counterstained with DAPI. Finally, fluorescence signals were detected and imaged using a confocal laser-scanning microscope (Carl Zeiss).

### Isolation of exosomes

Upon reaching 80% confluency, the cell cultures were washed with PBS and incubated for 48 h in freshly prepared complete medium containing exosome-free FBS. Exosomes were subsequently isolated from the CM through differential centrifugation. Specifically, the CM was initially centrifuged at 300*g* for 10 min, followed by 2,000*g* for 20 min at 4 °C to remove cells, and subsequently filtered through a 0.22-μm filter to eliminate cell debris. The exosomes were pelleted by ultracentrifugation at 100,000*g* for 90 min, resuspended in PBS, and then subjected to a second ultracentrifugation at 100,000*g* for 90 min. The size and concentration of the exosomes were measured using the qNano system (Izon Science, New Zealand), after which the exosomes were prepared for RNA/protein extraction or cell treatment.

### Exosomes labeling and tracing

The isolated exosomes were labeled with the lipophilic dye DiO (Invitrogen) following the manufacturer’s protocol. Subsequently, the labeled exosomes were washed with PBS, centrifuged and then incubated for 48 h with BLCA cells that had been pre-stained with the membrane dye Dil (Invitrogen) to assess exosome uptake. To examine the transfer of exosomal miRNA, CAFs were transfected with Cy5-labeled miR-1910-5p. These Cy5-miR-1910-5p-expressing CAFs were then co-cultured with BLCA cells for 48 h. The nuclei were stained with DAPI. Finally, the internalization of exosomes or exosomal miR-1910-5p was detected using a confocal laser-scanning microscope (Carl Zeiss).

### Luciferase reporter assay

Cells were transfected with luciferase reporter constructs containing either the wild-type (WT) or a mutated version of the FAP promoter or the CTDNEP1 3′UTR. For validation of FAP promoter activity, cells were co-transfected with either an empty vector or an MYC overexpression plasmid. For validation of CTDNEP1 3′UTR targeting, cells were co-transfected with either an empty vector, miR-1910-5p mimic or miR-1910-5p inhibitor. After 48 h of transfection, luciferase activities were measured using the Dual-Luciferase Reporter Assay Kit (Promega) in accordance with the manufacturer’s protocol. Firefly luciferase activity was normalized to Renilla luciferase activity to account for transfection efficiency.

### ChIP

Chromatin immunoprecipitation (ChIP) assay was conducted using the SimpleChIP Enzymatic Chromatin IP Kit (Cell Signaling Technology) according to the manufacturer’s instructions. Chromatin extracted from cells was first used to determine the total DNA input. Subsequently, the chromatin was incubated overnight with either specific antibodies or control IgG antibodies. Following immunoprecipitation, ChIP DNA was subjected to washing and purification steps as outlined in the protocol. The purified samples were then analyzed by qPCR. The primers and antibodies used are detailed in Supplementary Table 1 and Supplementary Table 2, respectively.

### Biotin miRNA pull-down

The cell lysates and exosomal lysates derived from CAFs were incubated overnight at 4 °C with 100 pmol of either synthetic single-stranded miR-1910-5p or its mutated counterpart, both of which were biotinylated. Subsequently, agarose beads (Invitrogen) were introduced to each binding reaction and further incubated at 4 °C for 4 h. The precipitates were then washed five times and subjected to SDS buffer boiling before being analyzed by western blot analysis.

### RIP

The Magna RNA immunoprecipitation (RIP) RNA-Binding Protein Immunoprecipitation Kit (Millipore) was utilized to perform the RIP assay. In brief, cells were collected and lysed in RIP lysis buffer, which was supplemented with protease and RNase inhibitors. The resulting protein extract was incubated overnight at 4 °C with an anti-RBMX antibody or a control IgG. Following incubation, the beads were washed, and the bead-bound immunoprecipitated was digested with proteinase K. RNA was then purified using a phenol-chloroform–isoamyl alcohol mixture and subsequently analyzed by qPCR. RNA levels were normalized to the input control. The fold enrichment of miR-1910-5p was calculated as a percentage of the input RNA and compared with the IgG isotypic control.

### Mice model

To investigate the functional role of WNT7A in lung metastasis models, stable human BLCA cell lines with either overexpression or depletion of WNT7A were intravenously injected into the tail veins of 4-week-old male BALB/c nude mice. A total of 6 weeks post injection, the mice were euthanized, and the lung tissues were collected, photographed and subsequently subjected to H&E and IHC analysis. To further examine the effect of exosomes on metastasis in vivo, stable murine BLCA MB49 cells overexpressing WNT7A or control cells were intravenously injected into the tail veins of 4-week-old male C57BL/6 mice. A total of 7 days after injection, mice were intraperitoneally injected with PBS or GW4869 (2 mg/kg) every other day. On day 25, the mice were killed, and lung tissues were collected, photographed and subsequently subjected to H&E and IHC analysis. Animal experiments were performed in accordance with protocols approved by the Ethics Committees of Shanghai General Hospital.

### Statistical analyses

Statistical analyses were performed using GraphPad Prism 10.0 (GraphPad Software). Data were presented as means ± standard deviation (s.d.). The significance between two or more groups was analyzed selectively by Student’s *t*-test or one-way ANOVA. Pearson correlation analysis was performed to determine the correlation between two variables. *P* < 0.05 was the threshold of significance.

## Results

### CAFs are enriched in the niche of lung metastases from BLCA

**Fig. 1:**
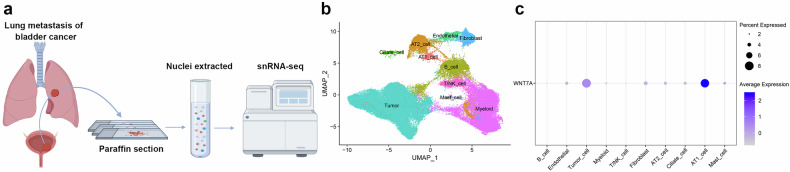
Identifying infiltrated cell types in lung metastases from BLCA. **a** Workflow of the sample preparation and sequencing. **b** Uniform manifold approximation and projection clustering of single-cell expression profiles. **c** Dot plot of WNT7A expression across different cell types.

To gain deeper insights into the microenvironmental characteristics of lung metastases from BLCA, we performed snRNA-seq on lung metastatic tissues from six patients with BLCA (Fig. 1a). Clustering analysis revealed the presence of not only tumor cells and immune cells but also a substantial enrichment of fibroblasts within the metastatic lesions. Notably, comparative analysis revealed a marked enrichment of CAFs in metastatic samples compared with control lungs (Fig. 1b and Supplementary Fig. 1a–d). This finding indicated that CAFs might play a critical role in modulating the microenvironment of lung metastases. The accumulation of CAFs in metastatic lesions is potentially closely associated with enhanced tumor cell migration, increased invasiveness and immune evasion, thereby facilitating tumor progression. Furthermore, our analysis demonstrated that WNT7A was highly expressed in tumor cells within metastatic lesions compared with other cell types (Fig. 1c).

### Tumor-derived WNT7A regulates fibroblasts activation

Based on the enrichment of CAFs in metastatic lesions and the high expression of WNT7A in tumor cells revealed by snRNA-seq, we hypothesized that WNT7A derived from tumor cells within the microenvironment of the metastatic foci might stimulate the activation of pulmonary fibroblasts into CAFs. To substantiate this hypothesis, a dual-source stimulation strategy was implemented: (1) endogenous WNT7A, a Transwell co-culture system was established, comprising BLCA cells and pulmonary fibroblasts, to replicate the tumor microenvironment with naturally secreted WNT7A; (2) exogenous WNT7A, direct stimulation was conducted using gradient concentrations of recombinant human WNT7A protein (rhWNT7A). The regulatory effects of WNT7A on fibroblast activation were comprehensively assessed by examining the expression of CAFs markers, including α-SMA and FAP, as well as alterations in cellular contractility.

**Fig. 2:**
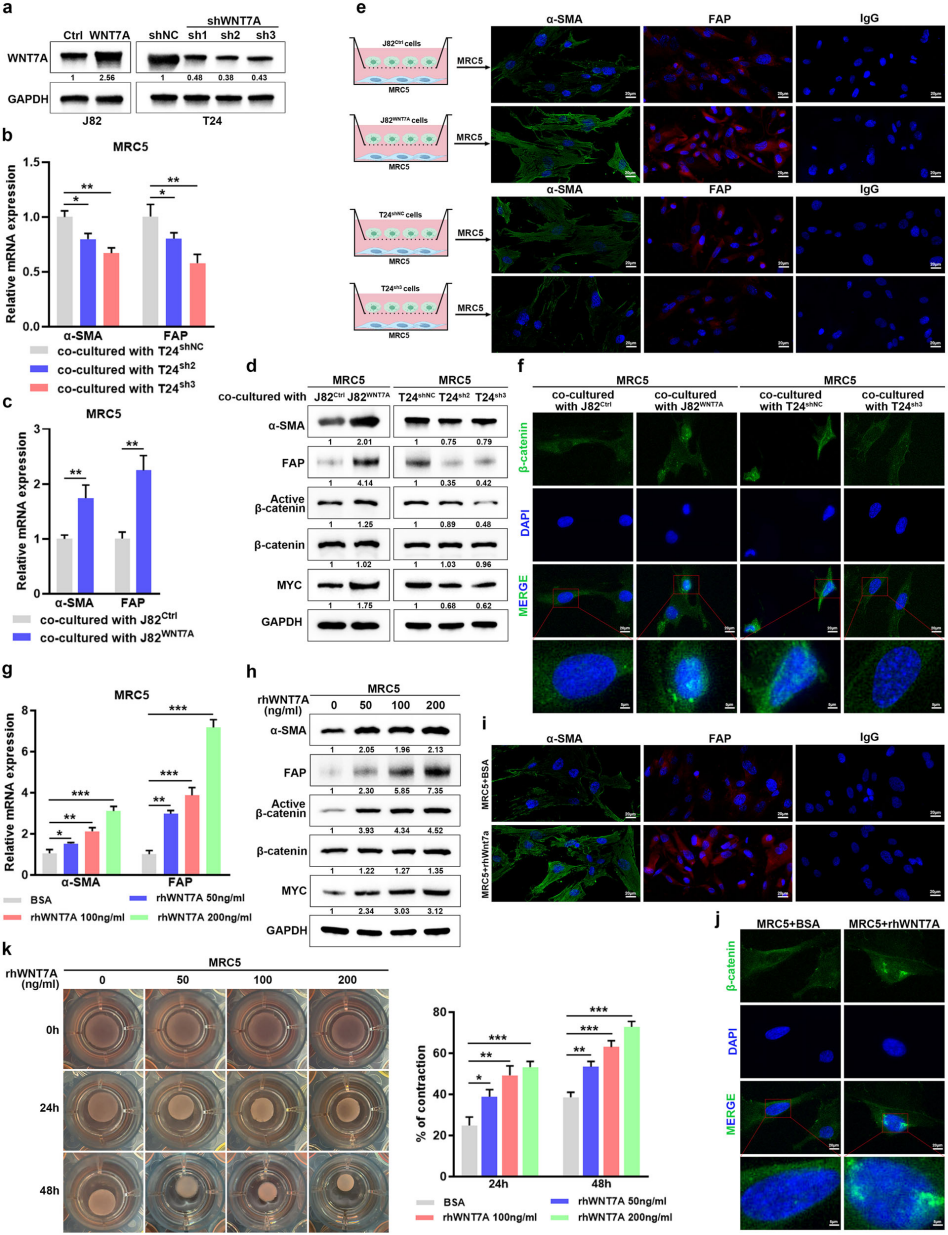
Tumor-derived WNT7A activates fibroblasts. **a** Western blot analysis confirmed overexpression of WNT7A in J82 cells and knockdown of WNT7A in T24 cells. **b** qPCR analysis of α-SMA and FAP expression in MRC5 after co-culture with WNT7A-knockdown T24 cells. **c** qPCR analysis of α-SMA and FAP expression in MRC5 after co-culture with WNT7A-overexpressing J82 cells. **d** Western blot analysis of specific proteins in MRC5 after co-culture with WNT7A-overexpressing J82 cells or WNT7A-knockdown T24 cells. **e** Immunofluorescence analysis of α-SMA and FAP expression in MRC5 after co-culture with WNT7A-overexpressing J82 cells or WNT7A-knockdown T24 cells. Isotype IgG was used as a control. **f** The localization of β-catenin in MRC5 after co-culture with WNT7A-overexpressing J82 cells or WNT7A-knockdown T24 cells. **g** qPCR analysis of α-SMA and FAP expression in MRC5 after stimulation with rhWNT7A. **h** Western blot analysis of specific proteins in MRC5 after stimulation with rhWNT7A. **i** Immunofluorescence analysis of α-SMA and FAP expression in MRC5 after stimulation with rhWNT7A. Isotype IgG was used as a control. **j** The localization of β-catenin in MRC5 after stimulation with rhWNT7A. **k** MRC5-mediated contraction of collagen matrices was evaluated after stimulation with rhWNT7A. The statistical data are presented as mean ± s.d., and the error bars represent the means of three independent experiments. *P < 0.05, **P < 0.01, ***P < 0.001.

To select appropriate BLCA cell lines for functional studies, we analyzed WNT7A expression across multiple lines. J82 cells, which exhibited relatively low WNT7A expression, were chosen for stable overexpression, while T24 cells, characterized by relatively high endogenous WNT7A levels, were selected for stable knockdown (Fig. 2a and Supplementary Fig. 2a). ELISA assays confirmed that WNT7A secretion was significantly increased in J82 cells overexpressing WNT7A and markedly decreased in T24 cells with WNT7A knockdown (Supplementary Fig. 2b). Subsequently, the established stable cell lines with either WNT7A overexpression or knockdown were independently co-cultured with MRC5 cells, a human embryonic lung fibroblast cell line designated as NFs in the present study. Quantitative analysis demonstrated a significant upregulation of α-SMA and FAP expression in MRC5 fibroblasts following co-culture with WNT7A-overexpressing J82 cells, whereas a marked downregulation of these CAFs markers was observed upon co-culture with WNT7A-knockdown T24 cells (Fig. 2b–e). Notably, the expression levels of activated β-catenin, a pivotal molecule in the Wnt/β-catenin signaling pathway, and its downstream transcriptional target MYC exhibited a consistent trend of change (Fig. 2d). Furthermore, immunofluorescence analysis revealed enhanced nuclear accumulation of β-catenin in MRC5 fibroblasts co-cultured with WNT7A-overexpressing J82 cells, in contrast to diminished nuclear localization following co-culture with WNT7A-knockdown T24 cells (Fig. 2f).

To further investigate the functional role of WNT7A in fibroblast activation, MRC5 cells were treated with rhWNT7A. Functional assays demonstrated that rhWNT7A stimulation enhanced the migratory and invasive capacities of MRC5 fibroblasts (Supplementary Fig. 3a,b). Furthermore, rhWNT7A stimulation led to increased expression of α-SMA and FAP in MRC5 cells (Fig. 2g–i). Mechanistically, rhWNT7A stimulation increased the protein levels of activated β-catenin and MYC (Fig. 2h) and promoted nuclear translocation of β-catenin in MRC5 cells (Fig. 2j). Given the well-documented association between fibroblast activation and enhanced extracellular matrix remodeling^19,20^, we subsequently examined the effect of rhWNT7A on collagen gel contraction, a hallmark of CAFs functionality. Quantitative analysis revealed that rhWNT7A treatment significantly enhanced the contractile capacity of MRC5 fibroblasts (Fig. 2k). Collectively, these findings suggest that WNT7A promotes the phenotypic transformation of MRC5 into CAFs, potentially through the Wnt/β-catenin signaling pathway.

### WNT7A facilitates fibroblasts activation via transcriptional upregulation of α-SMA and FAP through Wnt/β-catenin pathway

**Fig. 3:**
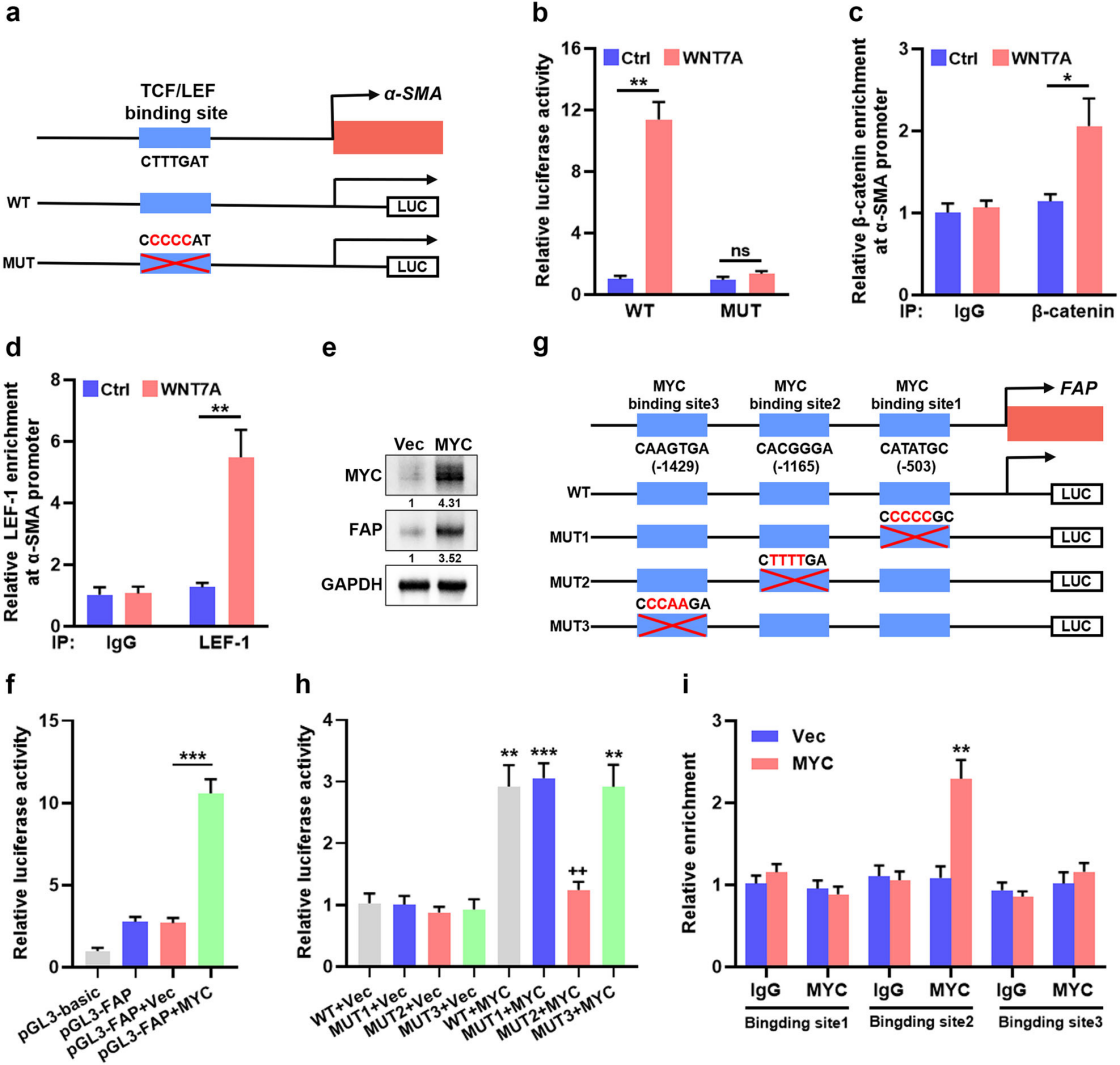
WNT7A transcriptionally activates the expression of α-SMA and FAP via the Wnt/β-catenin pathway. **a** Schematic representation of TCF/LEF binding site in α-SMA promoter. **b** Luciferase activity in MRC5 cells transfected with luciferase reporter vectors containing WT or TCF/LEF mutant sequences of α-SMA promoter and stimulated with or without WNT7A. **c** **d** ChIP analysis was performed using β-catenin or LEF-1 antibodies, and α-SMA promoter enrichment was detected by qPCR. **e** Western blot analysis of FAP expression in MYC-overexpressing MRC5 cells. **f** Luciferase activity in MRC5 cells transfected with luciferase reporter vectors containing FAP promoter fragment with overexpression of MYC. **g** Schematic representation of predicted MYC binding sites in FAP promoter. **h** Luciferase activity in MRC5 cell transfected with luciferase reporter vectors containing WT or mutant sequences of FAP promoter versus WT + vector, **P < 0.01, ***P < 0.001; versus WT-MYC, ^++^*P* < 0.01. **i** ChIP–qPCR analysis of MYC binding sites in FAP promoter. The statistical data are presented as mean ± s.d., and the error bars represent the means of three independent experiments. The statistical data are presented as mean ± s.d., and the error bars represent the means of three independent experiments. *P < 0.05, **P < 0.01, ***P < 0.001. ns, nonsignificant.

To further investigate the role of the Wnt/β-catenin signaling pathway in the transformation of NFs to CAFs, we initially examined whether the promoter regions of α-SMA and FAP contain binding sites for the TCF/LEF transcriptional complex, which is a downstream effector of the Wnt/β-catenin signaling pathway. This analysis was conducted using the consensus sequence 5′-CTTTGATG-3′, which is recognized by TCF/LEF^21^. Analysis revealed that the promoter of α-SMA contained the TCF/LEF binding sequence (Fig. 3a), whereas the promoter of FAP did not. Then we constructed a luciferase reporter driven by the α-SMA promoter, incorporating either the WT or a mutated version of the potential TCF/LEF binding sequence. As shown in Fig. 3b, treatment of MRC5 with WNT7A significantly enhanced the activity of the WT promoter but not the mutated promoter. ChIP assay using anti-β-catenin and anti-LEF-1 antibodies demonstrated that WNT7A treatment increased the binding of β-catenin and LEF-1 to the α-SMA promoter region in MRC5 cells (Fig. 3c, d). To further elucidate the transcriptional regulatory mechanisms underlying FAP expression, the databases hTFtarget, TFDB and GTRD were utilized to predict potential transcription factors that regulate FAP. The analysis identified 62 transcription factors that may be involved in the regulation of FAP expression (Supplementary Fig. 4a). Notably, MYC, a downstream target of the Wnt/β-catenin signaling pathway, emerged as a key candidate. Based on these findings, we hypothesize that WNT7A may activate FAP expression through the Wnt/β-catenin/MYC transcriptional axis. Supporting this, MYC overexpression in MRC5 cells markedly increased FAP levels at both mRNA and protein levels (Fig. 3e and Supplementary Fig. 4b). Further luciferase reporter assays confirmed MYC-mediated transcriptional activation of the FAP promoter in MRC5 cells (Fig. 3f), suggesting that MYC regulates FAP expression at the transcriptional level. Sequence analysis using the JASPAR database identified three potential MYC binding sites within the FAP promoter region (Fig. 3g and Supplementary Fig. 4c). Subsequent site-directed mutagenesis experiments revealed that binding site 2 was essential for MYC-induced transactivation of FAP (Fig. 3h). ChIP assay further confirmed the direct binding of MYC to the FAP promoter in MRC5 cells (Fig. 3i). Taken together, these results suggested that WNT7A transcriptionally activates the expression of α-SMA and FAP through the Wnt/β-catenin pathway, contributing to the activation of NFs to CAFs.

### WNT7A activates fibroblasts in vivo to remodel metastatic niche

**Fig. 4:**
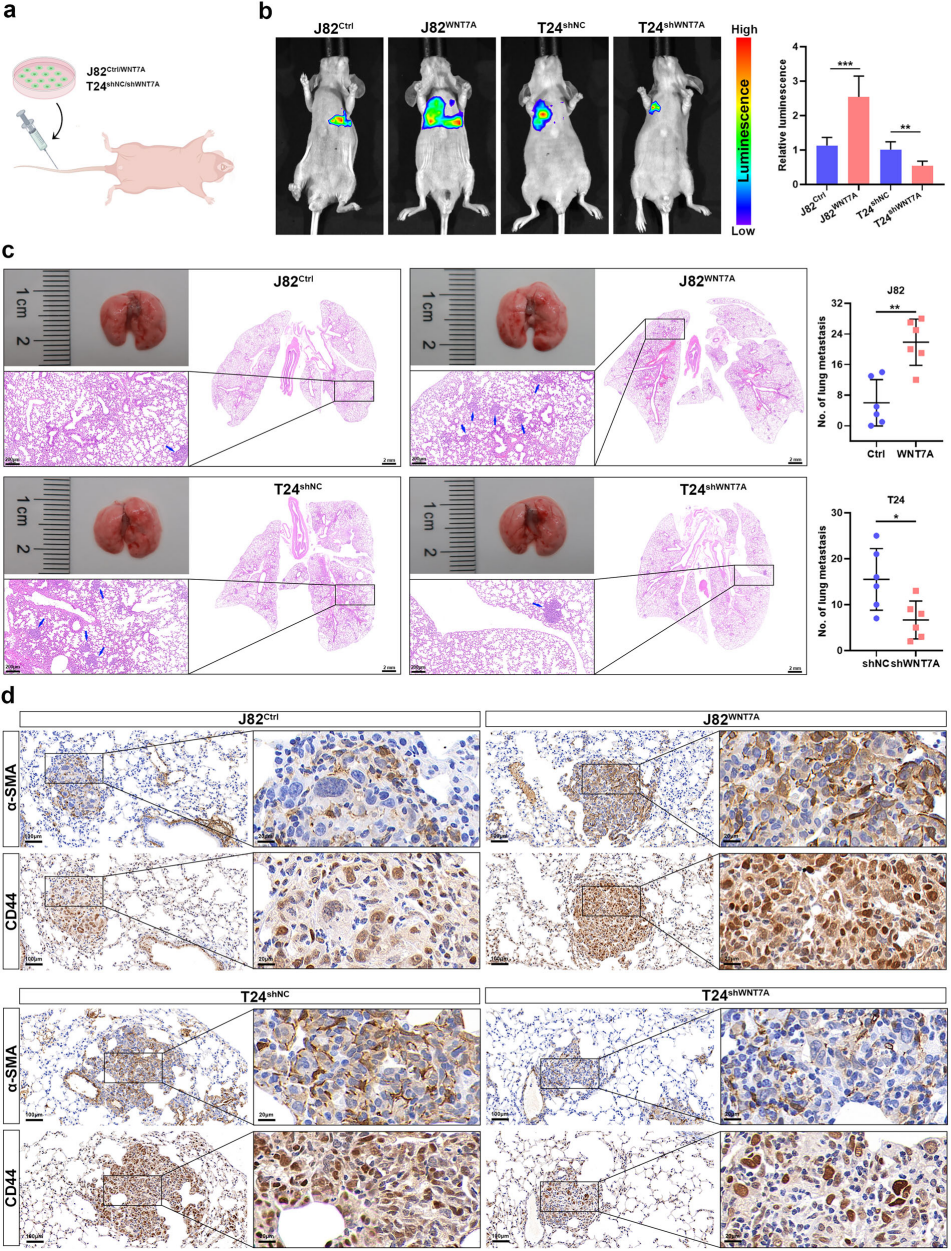
WNT7A activates lung fibroblasts to reprogram metastatic niches. **a** A schematic diagram of tail vein injection with WNT7A-modulated BLCA cells. **b** Representative images and quantification of luminescence in tail vein-injected mice. **c** The metastatic nodules in the lungs were sectioned and counted. Scale bars, 2 mm and 200 μm. **d** Representative IHC staining for α-SMA and CD44 in lung metastatic nodules from the WNT7A overexpression and silencing groups were presented. Scale bars, 100 and 20 μm. The statistical data are presented as mean ± s.d., and the error bars represent the means of six independent biological mouse samples. *P < 0.05, **P < 0.01, ***P < 0.001.

To elucidate the mechanistic role of WNT7A in pulmonary metastasis of BLCA, we established a mouse model of BLCA lung metastasis through tail vein injection of BLCA cells (Fig. 4a). It was observed that WNT7A overexpression significantly promoted lung metastasis of BLCA cells, whereas WNT7A knockdown markedly attenuated this effect (Fig. 4b,c). In the lung metastases, a marked enrichment of CAFs characterized by α-SMA positivity and BLCA stem cells marked by CD44 positivity was observed in the overexpression group compared with the control group. Conversely, the knockdown group showed the opposite results (Fig. 4d and Supplementary Fig. 5a, b). Furthermore, in patient-derived BLCA lung metastases, CAFs (α-SMA-positive) and BLCA stem cells (CD44-positive) in the infiltrating tumor tissue were found adjacent to each other, and even encapsulated, forming a metastatic niche in lung metastasis (Supplementary Fig. 6a). These results indicated that WNT7A promotes fibroblast activation in vivo, facilitating the reprogramming of the metastatic niche.

### CAFs-derived exosomes enhance BLCA cells malignancy

**Fig. 5:**
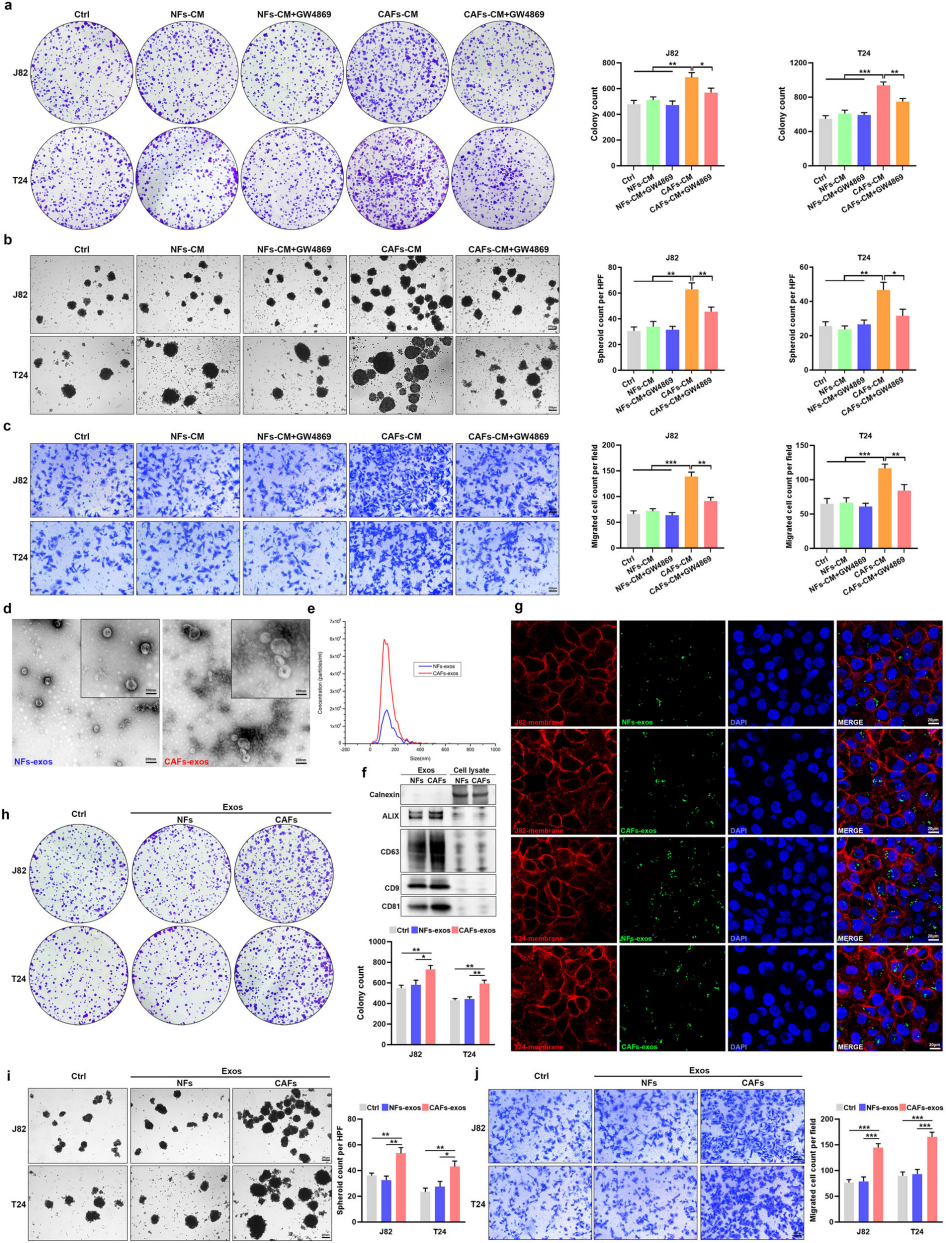
CAFs-derived exosomes promote the proliferation, stemness and migration of BLCA cells. **a** The impact of CM from NFs and CAFs, with or without the exosome inhibitor GW4869, on the proliferative capacity of BLCA cells. **b** The impact of CM from NFs and CAFs, with or without the exosome inhibitor GW4869, on the sphere-forming ability of BLCA cells. Scale bars, 200 μm. **c** The impact of CM from NFs and CAFs, with or without the exosome inhibitor GW4869, on the migratory ability of BLCA cells. Scale bars, 200 μm. **d** **e** Exosomes derived from NFs and CAFs were detected by electron microscopy **d** and Nanosight particle tracking analysis (**e**). Scale bar, 100 nm (top) and 200 nm (bottom). **f** Western blot analysis of exosomal markers ALIX, CD63, CD9 and CD81 in isolated exosomes and calnexin expression in cell lysate as the control. **g** Visualization of DiO-labeled exosome (green) delivery to Dil-labeled BLCA cells (red) by confocal microscopy. Scale bar, 20 μm. **h** The impact of exosomes from NFs and CAFs on the proliferative capacity of BLCA cells. **i** The impact of exosomes from NFs and CAFs on the the sphere-forming ability of BLCA cells. Scale bars, 200 μm. **j** The impact of exosomes from NFs and CAFs on the migratory ability of BLCA cells. Scale bars, 200 μm. The statistical data are presented as mean ± s.d., and the error bars represent the means of three independent experiments. *P < 0.05, **P < 0.01, ***P < 0.001.

In the microenvironment of lung metastases of BLCA, since WNT7A derived from BLCA cells could activate NFs into CAFs, what biological effects will the activated CAFs have on the BLCA cells metastasized to the lung? To further investigate the biological effects of WNT7A-induced activated CAFs on BLCA cells, we collected CM from CAFs to culture BLCA cells. Then, colony formation, spheroid formation and Transwell migration assays were applied to examine the effects of CAFs-CM on BLCA cells. The results revealed that, in comparison to NFs-CM, CAFs-CM markedly enhanced the proliferation, stemness and migration of BLCA cells (Fig. 5a–c). Exosomes, nanometric membrane vesicles secreted by almost all types of cells, playing a crucial role in intercellular communication^9,22^. As CAFs-CM promoted BLCA cells malignancy, we tried to investigate whether CAFs-derived exosomes might contribute to this effect. Fortunately, we found that pharmacologically blocking the exosome secretion of CAFs by GW4869 or physically removing exosomes from CAFs-CM by ultracentrifugation markedly reduced the ability of CAFs-CM to enhance BLCA cells malignancy (Fig. 5a–c and Supplementary Fig. 7a–c).

We then isolated and purified exosomes from the conditioned media of NFs and CAFs using the standard ultracentrifugation methods. The cup-shaped morphology, size and number of the isolated exosomes were subsequently characterized through electron microscopy and Nanosight particle tracking analysis (Fig. 5d, e). Western blot analysis verified the presence of exosomal markers ALIX, CD63, CD9 and CD81 in the isolated exosomes, whereas the endoplasmic reticulum membrane marker calnexin was not detected (Fig. 5f). To investigate exosome delivery, exosomes derived from NFs and CAFs were labeled with DiO and incubated with recipient BLCA cells that had been pre-labeled with Dil. Following incubation, confocal imaging revealed the presence of DiO-positive puncta within the recipient cells (Fig. 5g), indicating successful transfer of labeled exosomes from fibroblast cells to BLCA cells. Moreover, compared with exosomes derived from NFs, exosomes derived from CAFs could augment the proliferative capacity, tumorigenicity and migratory ability of BLCA cells (Fig. 5h–j). Collectively, these data indicated that exosomes released by CAFs enhanced malignancy of recipient BLCA cells.

### Exosome inhibitor inhibits lung metastasis of BLCA cells in vivo

**Fig. 6:**
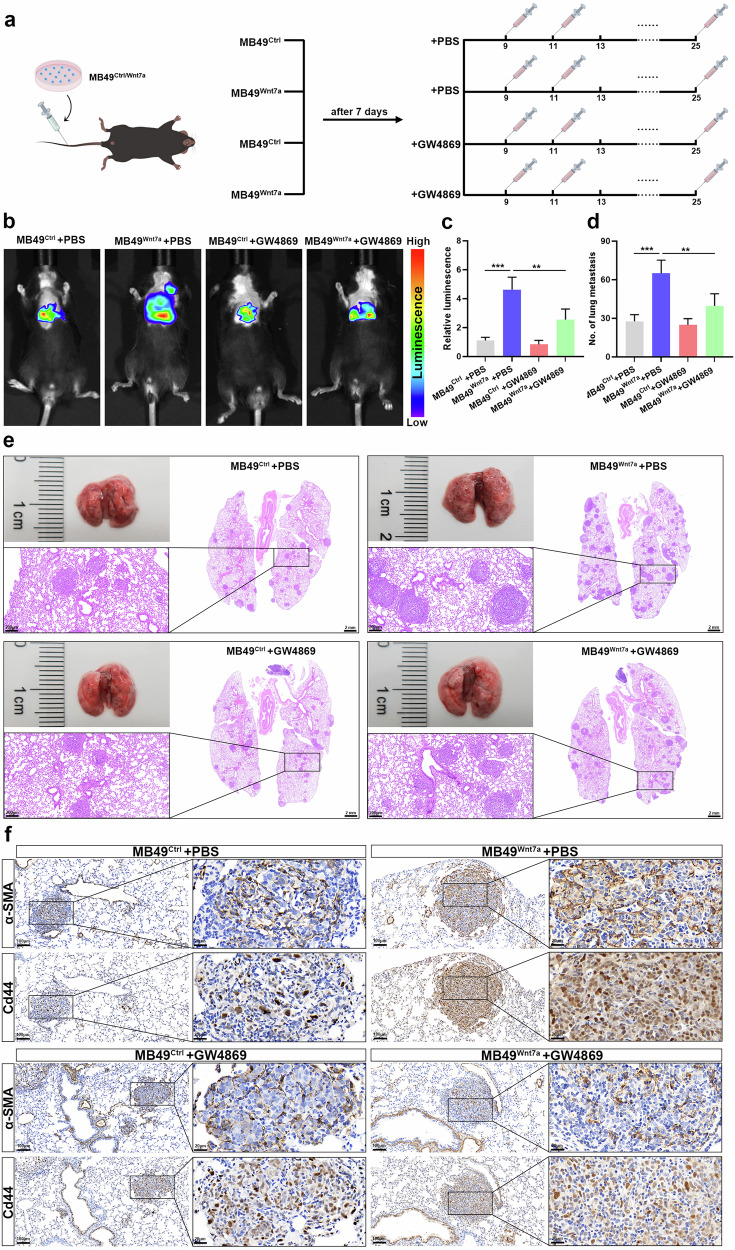
Exosome inhibitor suppresses lung metastasis of BLCA cells in vivo. **a** Schematic diagram of the mice tail vein metastasis model. **b** **c** Representative images (**b**) and quantification of luminescence (**c**) in tail vein-injected mice. **d**, **e** The metastatic nodules in the lungs were sectioned (**e**) and counted (**d**). Scale bars, 2 mm and 200 μm. **f** Representative IHC staining for α-SMA and Cd44 in lung metastatic nodules from tail vein metastasis model were presented. Scale bars, 100 and 20 μm. The statistical data are presented as mean ± s.d., and the error bars represent the means of six independent biological mouse samples. **P < 0.01, ***P < 0.001.

To determine whether CAFs-derived exosomes affected BLCA cells lung metastasis in vivo, we established a lung metastasis model by injecting Wnt7a-overexpressing MB49 and control cells separately via the tail vein. Consistently, in vitro experiments confirmed that the overexpression of Wnt7a enhanced the proliferation and migration capabilities of MB49 cells (Supplementary Fig. 8a–c). A total of 7 days after the injection, the mice received intraperitoneal injection of 2 mg/kg GW4869 or PBS at 2-day intervals (Fig. 6a). It was observed that the overexpression of Wnt7a enhanced the lung metastasis of MB49 cells, whereas treatment with GW4869 resulted in a reduction in both luminescence intensity and the number of nodules in lung metastatic tumors (Fig. 6b–e). In addition, a marked enrichment of CAFs characterized by α-SMA positivity and BLCA stem cells marked by Cd44 positivity was observed in the overexpression group within the lung metastatic lesions. Furthermore, GW4869 treatment led to a reduction in the expression levels of α-SMA and Cd44 (Fig. 6f and Supplementary Fig. 8d,e). These results indicated that the exosome inhibitor GW4869 effectively suppressed lung metastasis of BLCA cells in vivo.

### Exosomal transfer of miR-1910-5p from CAFs to BLCA cells

**Fig. 7:**
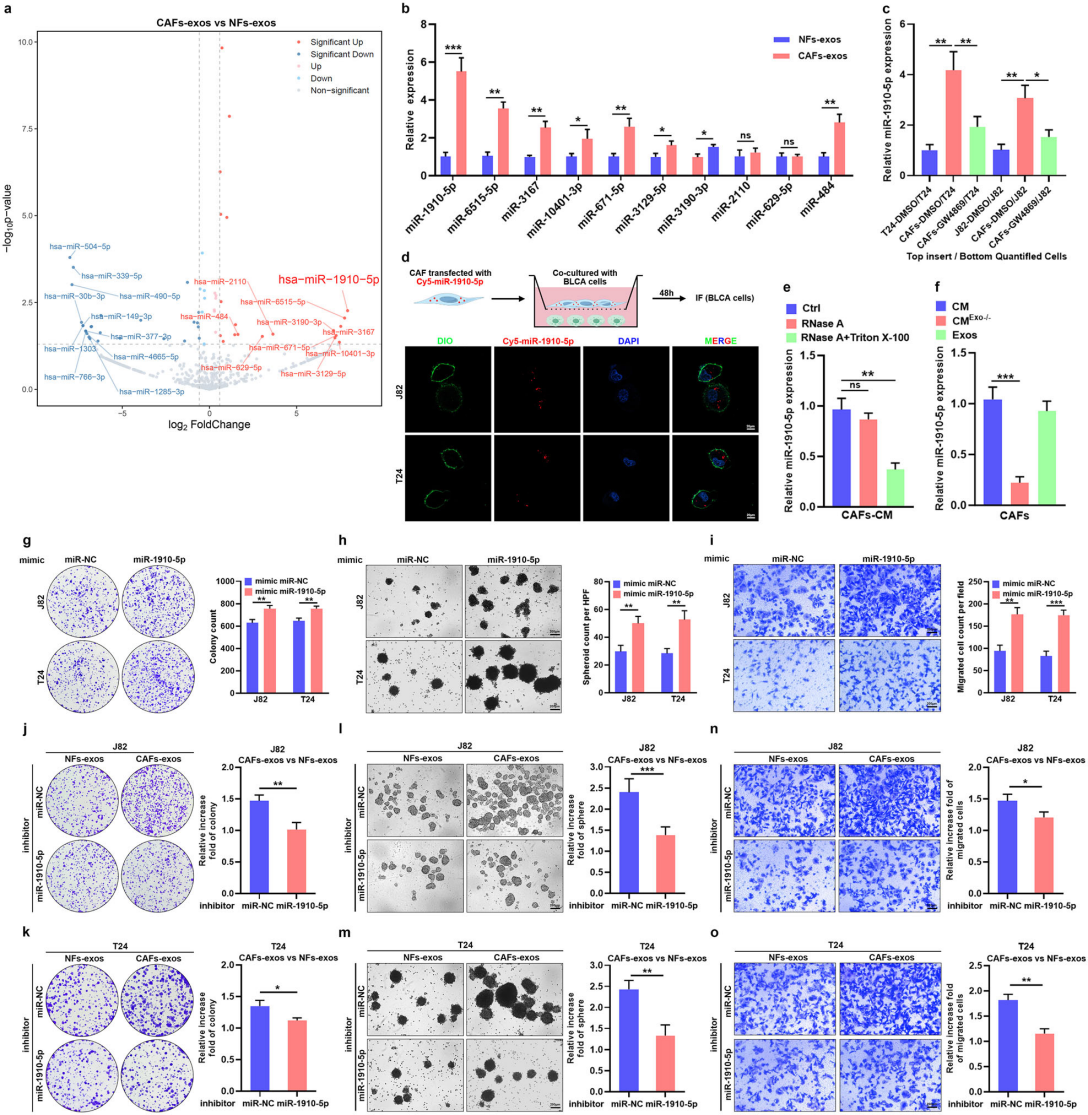
Exosomal transfer of miR-1910-5p from CAFs to BLCA cells. **a** Microarray analysis of exosomal miRNAs from NFs and CAFs were presented in a volcano plot. **b** qPCR analysis of the expression levels of the top ten differentially expressed miRNAs expression with the most significantly upregulated changes. **c** qPCR analysis of miR-1910-5p expression in BLCA cells after co-culture with DMSO-treated cells. **d** CAFs transiently transfected with Cy5-tagged miR-1910-5p were co-cultured with BLCA cells. Fluorescence microscopy was used to detect the red fluorescent signals in BLCA cells. Scale bar, 20 μm. **e** qPCR analysis of miR-1910-5p expression in CAFs-CM treated with RNase A alone or combined with Triton X-100. **f** qPCR analysis of miR-1910-5p expression in whole CM, exosome-depleted CM and exosomes derived from CAFs. **g**–**i** Effect of miR-1910-5p mimic on BLCA cells proliferation (**g**) stemness (**h**) and migration (**i**). Scale bar, 200 μm. **j–o** Proliferative capacity (**j** and **k**) tumorigenicity (**l** and **m**) and migratory ability (**n** and **o**) comparison of BLCA cells treated with exosomes derived from NFs or CAFs and transfected with miR-1910-5p inhibitor or negative control. Scale bar, 200 μm. The statistical data are presented as mean ± s.d., and the error bars represent the means of three independent experiments. *P < 0.05, **P < 0.01, ***P < 0.001. ns, nonsignificant.

We subsequently investigated the role of CAFs-derived exosomes in promoting malignancy in recipient BLCA cells. Given the abundance and importance of miRNAs encapsulated within exosomes in cell–cell communication^23^, we hypothesized that exosomal miRNAs originating from CAFs contribute to the aggressiveness of BLCA cells. To pinpoint the specific miRNAs involved, we performed microarray analyses to generate miRNA profiles from NFs- and CAFs-derived exosomes. The differentially expressed miRNAs were presented as heatmap in Fig. 7a, with miR-1910-5p demonstrating the most pronounced change in expression. Further GO and KEGG functional analysis indicated that the enrichment of differentially expressed miRNAs was closely associated with molecular pathways related to metastasis, such as regulation of epithelial to mesenchymal transition and cell adhesion molecule binding (Supplementary Fig. 9a,b). The top ten miRNAs with the most significantly upregulated were selected for qPCR validation (Fig. 7b). Furthermore, a notable increase in miR-1910-5p expression was observed in BLCA cells following incubation with CAFs-derived exosomes (Supplementary Fig. 10a,b). These results indicated that CAFs within the tumor microenvironment might enhance miR-1910-5p expression level in BLCA cells via direct transfer of miR-1910-5p.

To ascertain the direct transfer of miR-1910-5p from CAFs to tumor cells via exosomes, BLCA cells were cultured with either CAFs-CM or exosome-depleted CAFs-CM. Notably, BLCA cells cultured in CAFs-CM exhibited elevated levels of miR-1910-5p. Conversely, when exosomes were depleted from CAFs-CM using GW4869, the expression of miR-1910-5p in BLCA cells was markedly reduced (Fig. 7c). In addition, CAFs were transiently transfected with Cy5-tagged miR-1910-5p and co-cultured with BLCA cells for 48 h. Confocal microscopy revealed the presence of fluorescently tagged miR-1910-5p within the cancer cells, indicating that the transfer of miR-1910-5p from CAFs to BLCA cells via exosomes (Fig. 7d). Furthermore, the concentration of miR-1910-5p in CAFs-CM remained unchanged following treatment with RNase A, but significantly decreased when treated with both RNase A and Triton X-100 (Fig. 7e). This suggests that extracellular miR-1910-5p is primarily encapsulated within membrane structures rather than being directly released. Interestingly, the levels of miR-1910-5p were almost identical in both exosomes and the entire CAFs-CM (Fig. 7f). These results indicated that miR-1910-5p was transferred from CAFs to BLCA cells via exosomes.

### miR-1910-5p promotes BLCA cells malignancy

Having determined that BLCA cells could uptake CAFs-derived exosomal miR-1910-5p, we next aimed to examine whether miR-1910-5p might contribute to BLCA cell malignant progression. As a result, miR-1910-5p mimic markedly augmented the proliferative capacity, tumorigenicity and migratory ability of BLCA cells (Fig. 7g–i). Furthermore, the miR-1910-5p inhibitor was able to partially abrogate the effects of CAFs-derived exosomes in promoting the malignancy of BLCA cells (Fig. 7j–o). Thus, we concluded that miR-1910-5p could enhance BLCA cells malignancy.

### miR-1910-5p packaging into exosomes is mediated by RBMX

**Fig. 8:**
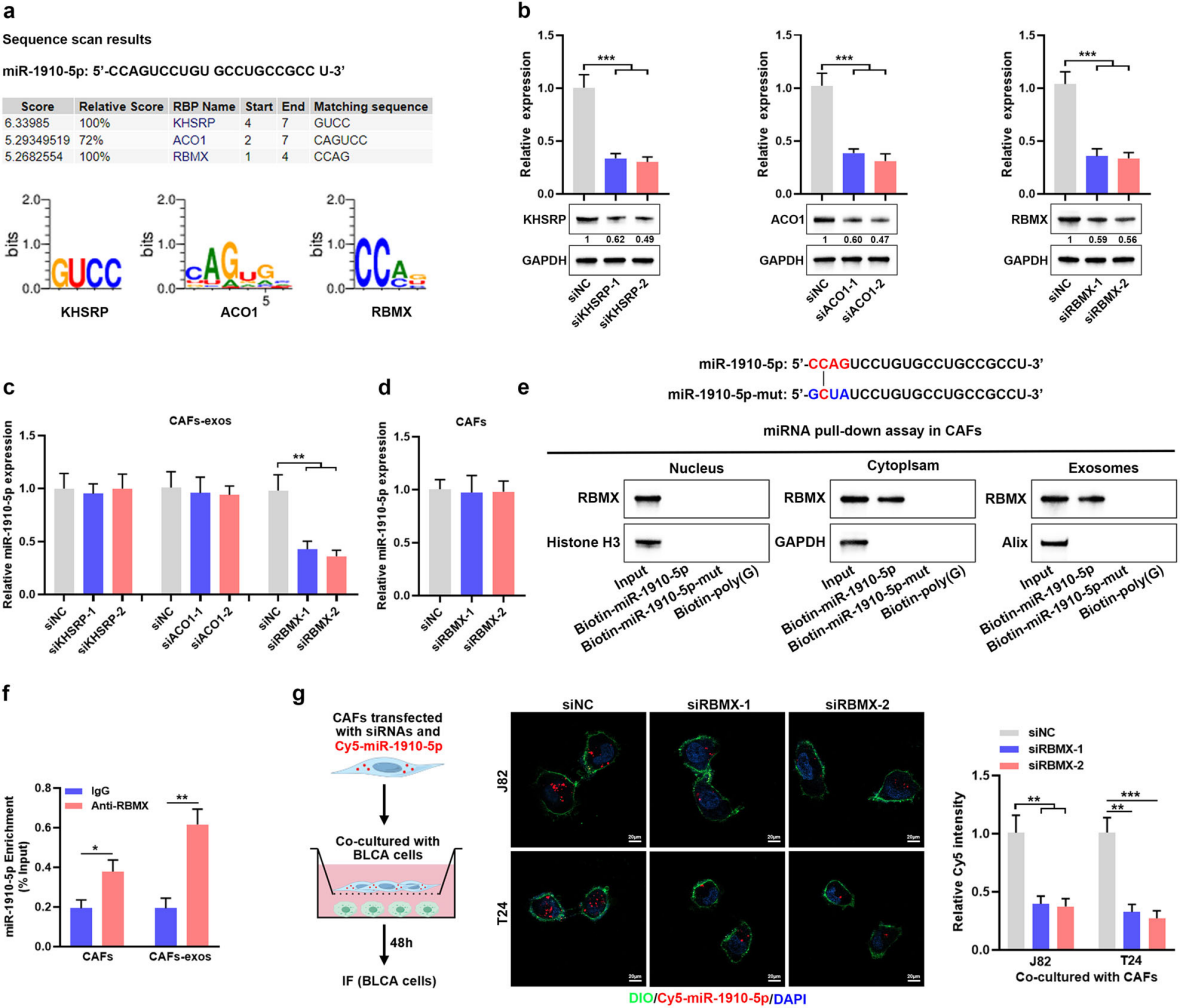
The RBMX mediates miR-1910-5p packaging into CAFs-derived exosomes. **a** RBPDB analysis of the specific interaction between miR-1910-5p and RBP motifs. **b** qPCR and western blotting detected KHSRP, ACO1 and RBMX expression levels in CAFs after transfection with the specific siRNAs. **c** qPCR analysis of miR-1910-5p expression in exosomes from CAFs transfected with specific siRNAs. **d**, qPCR analysis of miR-1910-5p expression in CAFs with RBMX silenced. **e** The miRNA pull-down experiment analyzed the interaction of miR-1910-5p and miR-1910-5p mutant with RBMX in the cytoplasm, nucleus and exosomal lysates of CAFs, biotinylated poly(G) was used as a negative control. **f** RIP assays with anti- RBMX antibody (or IgG as control) were performed on the cell or exosomal lysates from CAFs. **g** BLCA cells were co-cultured with CAFs concurrently transfected with Cy5-miR-1910-5p and specific siRNAs targeting RBMX for 48 h. Fluorescence microscopy was used to detect red fluorescent signals in BLCA cells. Scale bar, 20 μm. The statistical data are presented as mean ± s.d., and the error bars represent the means of three independent experiments. *P < 0.05, **P < 0.01, ***P < 0.001.

To determine whether miR-1910-5p was selectively packaged into exosomes, we examined the specific interaction between the miR-1910-5p sequence and the motifs of RNA-binding proteins (RBPs). This analysis was conducted utilizing the RNA-Binding Protein Database (RBPDB, http://rbpdb.ccbr.utoronto.ca/; threshold 0.7)^24^. Figure 8a illustrates that the motifs of the top three RBPs possess specific binding sites for miR-1910-5. KHSRP, ACO1 and RBMX were respectively knocked down using siRNAs (Fig. 8b). Subsequent investigations demonstrated that specific knockdown of RBMX significantly reduced the abundance of miR-1910-5p in CAFs-derived exosomes, whereas the intracellular levels of miR-1910-5p remained unaffected (Fig. 8c, d). This finding suggests that RBMX primarily facilitates the selective incorporation of miR-1910-5p into exosomes rather than regulating its intracellular expression or stability. By contrast, knockdown of KHSRP and ACO1 had no significant impact on exosomal miR-1910-5p levels (Fig. 8c), ruling out their involvement in this process and further confirming the pivotal role of RBMX in miR-1910-5p sorting. Furthermore, miRNA pull-down assays revealed that RBMX interacted with miR-1910-5p in both the cytoplasm and exosomes but not in the nucleus (Fig. 8e). Notably, the binding capacity of RBMX was compromised when the CCAG sequence of miR-1910-5p was mutated (Fig. 8e). In addition, RIP assay was conducted on both cellular and exosomal lysates of CAFs. The results indicated a marked enrichment of miR-1910-5p in the RBMX antibody group compared with the IgG control group (Fig. 8f). Functionally, confocal analysis demonstrated that the transfer of Cy5-labeled miR-1910-5p from CAFs to BLCA cells via exosomes was diminished when CAFs were pre-transfected with RBMX-specific siRNAs (Fig. 8g). These data demonstrated that RBMX might play a crucial role in the packaging of miR-1910-5p into exosomes by binding to a specific motif located at the 5′ end of miR-1910-5p, indicating that miR-1910-5p was selectively sorted into exosomes derived from CAFs.

### Exosomal miR-1910-5p directly targets CTDNEP1 in BLCA cells

**Fig. 9:**
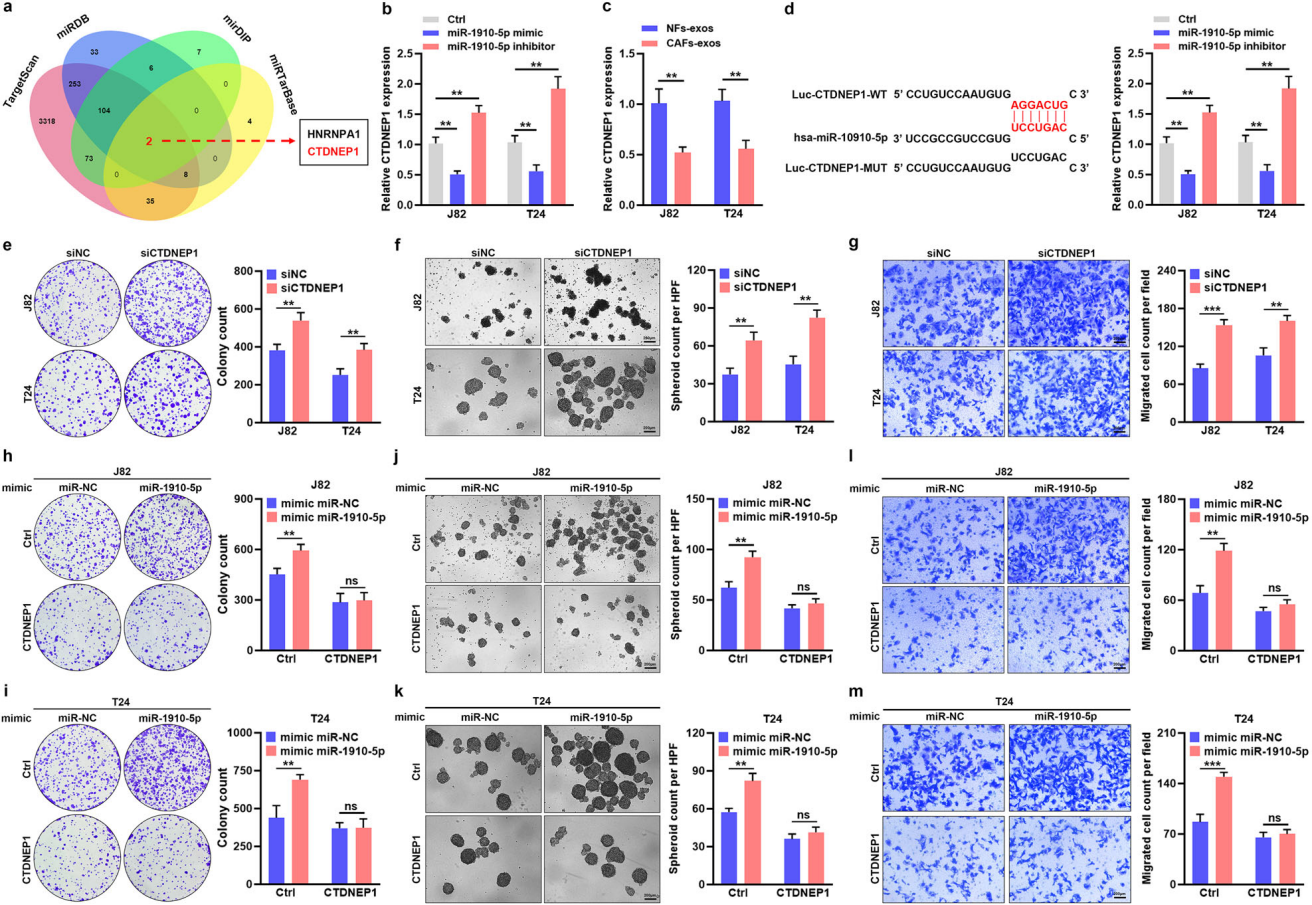
CTDNEP1 is the direct target of exosomal miR-1910-5p in BLCA cells. **a** Venn diagram of the miR-1910-5p putative candidate target genes predicted by TargetScan, miRDB, mirDIP and miRTarBase. **b** qPCR analysis of CTDNEP1 expression in BLCA cells after treated with miR-1910-5p mimic or inhibitor. **c** qPCR analysis of CTDNEP1 expression in BLCA cells after incubation with exosomes from NFs or CAFs. **d** Luciferase activity in cells co-transfected with Luc-CTDNEP1 WT or mutant sequence and miR-1910-5p mimic or inhibitor. **e**–**g** Effect of CTDNEP1 knockdown on BLCA cells proliferation (**e**) stemness (**f**) and migration (**g**). Scale bar, 200 μm. **h–m** miR-1910-5p effect on proliferative capacity (**h** in J82 and **i** in T24) tumorigenicity (**j** in J82 and **k** in T24) and migratory ability (**l** in J82 and **m** in T24) of BLCA cells in the presence of CTDNEP1 or not. Scale bar, 200 μm. The statistical data are presented as mean ± s.d., and the error bars represent the means of three independent experiments. **P < 0.01, ***P < 0.001. ns, nonsignificant.

To identify the target genes of exosomal miR-1910-5p in BLCA cells, the databases TargetScan, miRDB, mirDIP and miRTarBase were utilized. The analysis predicted HNRNPA1 and CTDNEP1 as potential downstream targets of miR-1910-5p (Fig. 9a). Data from TCGA was utilized to investigate the potential correlation between the expression of candidate downstream targets and miR-1910-5p. The analysis revealed an inverse association between the expression of CTDNEP1 and miR-1910-5p, whereas no significant association was observed for HNRNPA1 (Supplementary Fig. 11a,b). Notably, the miR-1910-5p mimic suppressed CTDNEP1 expression in BLCA cells, whereas the miR-1910-5p inhibitor produced the opposite effect (Fig. 9b and Supplementary Fig. 11c,d). As expected, exosomal miR-1910-5p derived from CAFs markedly inhibited CTDNEP1 expression in BLCA cells (Fig. 9c). However, neither miR-1910-5p mimic and miR-1910-5p inhibitor, nor exosomal miR-1910-5p derived from CAFs had a significant effect on the expression of HNRNPA1 (Supplementary Fig. 11e,f). Therefore, we speculated that CTDNEP1 was a potential target of miR-1910-5p. Dual-luciferase reporter assays demonstrated that miR-1910-5p binds to the 3ʹ-UTR of CTDNEP1, further indicating that CTDNEP1 was a direct target of miR-1910-5p (Fig. 9d). We then analyzed the functional roles of CTDNEP1 in BLCA cells. Silence of CTDNEP1 markedly promoted BLCA cell proliferation, stemness and migration (Fig. 9e–g and Supplementary Fig. 12a). Moreover, overexpression of CTDNEP1 could neutralize the effects of miR-1910-5p in promoting the malignancy of BLCA cells (Fig. 9h–m and Supplementary Fig. 12b). Consequently, we concluded that CTDNEP1 is a functional target gene of miR-1910-5p.

### The miR-1910-5p/CTDNEP1/MYC signaling axis regulates the colonization of BLCA cells in the lung

**Fig. 10:**
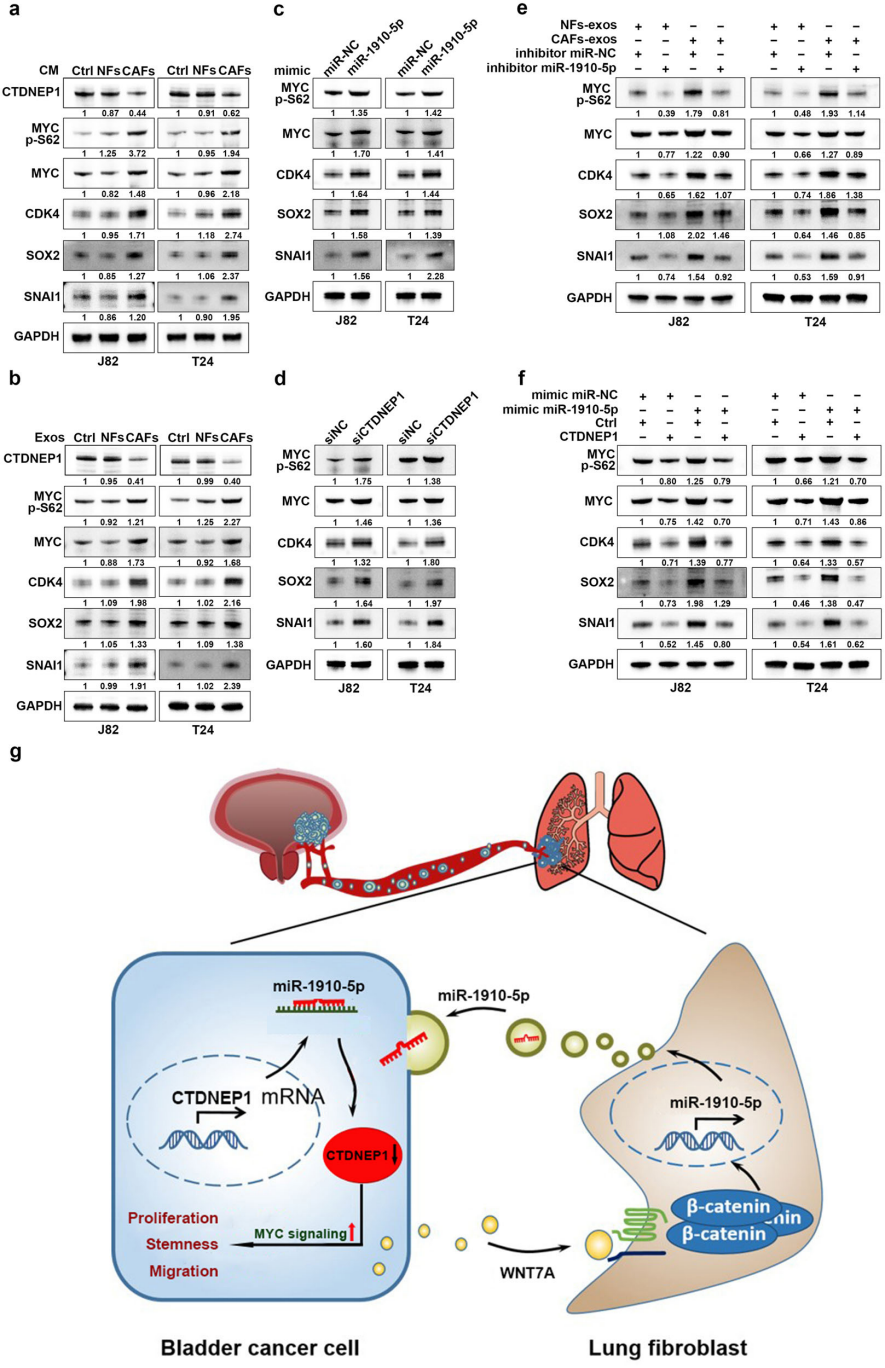
The miR-1910-5p/CTDNEP1/MYC signaling axis regulates the colonization of BLCA cells in the lung. **a** **b** Western blot analysis of specific proteins in BLCA cells treated with CM or exosomes from NFs or CAFs. **c** Effect of miR-1910-5p mimic on specific proteins expression in BLCA cells. **d** Effect of CTDNEP1 knockdown on specific proteins expression in BLCA cells. **e** **f** Western blot analysis of specific proteins in BLCA cells with indicated treatments. **g** Proposed mechanism of BLCA lung metastasis. Tumor-derived WNT7A stimulates CAFs activation, which in turn promotes exosomal transfer of miR-1910-5p to facilitate lung metastasis.

CTDNEP1, a CTD nuclear-envelope phosphatase, is known to interact with MYC and modulate its stability through dephosphorylation at serine 62 (S62). The inhibition or reduced expression of CTDNEP1 could prevent MYC dephosphorylation, thereby promoting its phosphorylation at S62, stabilizing the protein and enhancing its transcriptional activity^25^. The inverse relationship between MYC and CTDNEP1 suggested that the suppression of CTDNEP1 might contribute to the upregulation of MYC in the lung metastases of BLCA. Thus, we further investigated the regulatory relationship between CTDNEP1 and MYC and its downstream targets. Analysis of snRNA-seq revealed a differential expression profile between MYC and CTDNEP1 within the tumor microenvironment. Specifically, MYC exhibited higher expression levels compared with CTDNEP1 in tumor cells (Supplementary Fig. 13a). According to the western blot results, compared with the CM or exosomes derived from NFs, treatment of BLCA cells with CM or exosomes derived from CAFs resulted in decreased expression levels of CTDNEP1 (Fig. 10a, b). Conversely, the expression levels of pS62-MYC, MYC and its downstream targets, including CDK4, which regulates proliferation, SOX2, which regulates stemness, and SNAI1, which regulates migration were increased (Fig. 10a, b). Furthermore, miR-1910-5p expression or CTDNEP1 suppression also increased the protein levels of pS62-MYC, MYC and its downstream targets, including CDK4, SOX2 and SNAI1 (Fig. 10c, d). Moreover, the inhibition of miR-1910-5p could counteract the promotion of MYC expression and its downstream targets induced by CAFs-derived exosomes (Fig. 10e). In addition, the overexpression of CTDNEP1 could mitigate the effect of miR-1910-5p mimic, thereby reversing the enhancement of MYC expression and its downstream targets (Fig. 10f). To comprehensively elucidate the clinical relevance of the miR-1910-5p/CTDNEP1/MYC regulatory axis in BLCA pulmonary metastasis, FISH of miR-1910-5p was performed in combination with IHC staining for CTDNEP1 and MYC on human BLCA lung metastatic tissue sections. The metastatic lesions exhibited a coordinated pattern of high miR-1910-5p and MYC expression, along with consistently low CTDNEP1 expression (Supplementary Fig. 13b–e). Collectively, these results suggested that exosomal miR-1910-5p suppressed CTDNEP1 expression to activate MYC signaling to promote the colonization of BLCA cells in the lung.

## Discussion

Tumors pose a serious threat to human health, and metastasis is the primary cause of cancer-related mortality^26,27^. Therefore, elucidating the mechanisms of tumor metastasis and subsequently inhibiting it are crucial for effective cancer treatment. Tumor metastasis is widely recognized as a complex, multi-stage process involving several critical steps. This includes the detachment of tumor cells from the primary site, their subsequent migration and invasion into the lymphatic and circulatory systems and, ultimately, the colonization of distant organs, culminating in the formation of metastatic lesions^28–30^. Among these steps, the colonization of target organs is the most complex and critical rate-limiting step^3^. Consequently, the microenvironment at the sites of tumor cell colonization undergoes a series of molecular events that are associated with the establishment of tumor cells and the promotion of their survival. Increasing evidence confirms that tumor cells can remodel and condition the local environment of metastatic target organs by secreting soluble molecules and other substances, thereby creating a favorable metastatic niche for tumor cell colonization^31,32^. The establishment of metastatic niche represents an orchestrated multicellular process wherein CAFs serve as functional linchpins within the stromal microenvironment^33^. Emerging evidence positions CAFs as predominant cellular constituents in metastatic lesions and central signaling hubs coordinating tumor-stromal crosstalk through paracrine factor secretion, metabolic reprogramming and extracellular matrix remodeling^34–36^. It is widely accepted that CAFs originate from fibroblasts residing within or adjacent to the tumor^5,20^; however, the mechanisms underlying CAFs activation remain incompletely understood.

In our previous study, we revealed that the expression level of the Wnt family member WNT7A was significantly higher in BLCA tissues compared with adjacent normal bladder tissues. Moreover, high expression of WNT7A was positively correlated with metastasis and poor survival prognosis in BLCA. In vitro experiments demonstrated that WNT7A enhanced the proliferation and invasiveness of BLCA cells, while in vivo studies further confirmed that overexpression of WNT7A notably increased the pulmonary metastatic potential of BLCA cells^17^. However, the specific molecular mechanisms by which WNT7A promotes BLCA cells metastasis remain unclear. In this study, we further explored the role of WNT7A in the tumor microenvironment, particularly its function in promoting BLCA metastasis through the regulation of CAFs activation. We found that tumor-derived WNT7A transcriptionally upregulated the expression of CAFs markers α-SMA and FAP by activating the Wnt/β-catenin signaling pathway, thereby inducing the conversion of NFs into CAFs. This conversion not only altered the fibroblast phenotype but also enhanced their supportive role in BLCA progression, further promoting the malignant transformation of BLCA cells. Although WNT7A is a well-established canonical Wnt ligand capable of activating β-catenin signaling, it remained unclear whether WNT7A elicits a distinct fibroblast activation program compared with other Wnt ligands. Our snRNA-seq based ligand expression analysis revealed a predominant enrichment of WNT7 family ligands in the lung metastatic microenvironment, with WNT7B exhibiting relatively higher expression among Wnt ligands (Supplementary Fig. 14a,b). However, functional validation experiments demonstrated that WNT7A exerted a markedly stronger capacity to activate fibroblasts than WNT7B (Fig. 2g, h and Supplementary Fig. 14c,d). These findings indicate that the CAF activation program described here is not merely a nonspecific consequence of elevated Wnt signaling, but rather a specific mechanism driven predominantly by WNT7A.

Notably, compared with NFs-CM, CAFs-CM significantly increased the proliferation, stemness and migration of BLCA cells. This finding further confirmed the ability of CAFs to enhance the malignant phenotype of BLCA cells. Although these preliminary results highlight the crucial role of CAFs in BLCA progression, the precise molecular mechanisms underlying this process remain unclear. The mechanisms through which CAFs regulate malignant behaviors, including the secretion of chemokines, cytokines and growth factors, have been well-characterized^37–39^. Recent studies have further highlighted the critical role of CAFs-derived exosomes in cancer progression. Exosomes, which are small extracellular vesicles involved in intercellular communication, can transport proteins, lipids and nucleic acids, such as miRNAs, from CAFs to cancer cells, thereby influencing tumor dynamics^40,41^. miRNAs, as the primary RNA component of exosomes, are often dysregulated in various cancers and play a crucial role in modulating cancer progression and metastasis^13,14,42^. The CAFs-derived exosomal transfer of specific miRNAs, including miR-500a-5p^43^, miR-522^44^, miR-20a^45^ and miR-146a-5p^46^, has been demonstrated to substantially influence the interaction between cancer cells and the tumor microenvironment. In this study, we demonstrated that exosomes secreted by CAFs enhanced the malignancy of recipient BLCA cells. We also identified miR-1910-5p as a key exosomal cargo derived from CAFs that significantly promoted the proliferation, stemness and migration of BLCA cells. Notably, miR-1910-5p is an understudied miRNA in cancer research, with only one prior study reported its upregulation in plasma-derived extracellular vesicles from patients with head and neck squamous cell carcinoma^47^. The specific roles and mechanisms of miR-1910-5p in BLCA remain largely unexplored. In our investigation, by incubating BLCA cells with CAFs transfected with Cy5-labeled miR-1910-5p, we confirmed the transfer of miR-1910-5p into the cancer cells, leading to enhanced proliferation, stemness and migration. Crucially, we further conducted an in-depth analysis of the packaging mechanism of miR-1910-5p within exosomes. In the context of exosomal miRNA export, RBPs such as hnRNPA1, hnRNPA2B1 and hnRNPQ have been demonstrated to play pivotal roles by binding to specific motifs^44,48–50^. In addition to these hnRNPs, other proteins including FUS, Ago2 and Y-box protein 1 have been shown to be critically involved in miRNA trafficking within exosomes^51–53^. In this study, we demonstrate that RBMX specifically binds to miR-1910-5p via a distinct sequence at the 5′ end of the miRNA. This interaction facilitates the packaging of miR-1910-5p into exosomes, potentially offering a mechanism for the removal of miR-1910-5p within the metastatic niche during the treatment of lung metastases. However, the precise mechanisms underlying the packaging of miR-1910-5p into exosomes remain incompletely understood. Consequently, additional research is required to elucidate these processes more thoroughly in future studies.

Exosome-mediated transport is recognized as an effective mechanism for modulating cell signaling and biological functions in recipient cells. Our results revealed that exosomal miR-1910-5p, upon delivery from CAFs to BLCA cells, enhanced malignant properties by specifically targeting CTDNEP1. CTDNEP1 encodes a protein integral to the regulation of phosphatases associated with the nuclear envelope. Its primary function involves the dephosphorylation of proteins localized within the nuclear envelope and endoplasmic reticulum membranes, a process essential for maintaining various cellular activities^54^. Specifically, CTDNEP1 plays a crucial role in cell cycle regulation by counteracting mTOR kinase, thereby establishing a dephosphorylated pool of the phosphatidic acid phosphatase lipin 1 during interphase^55^. Moreover, CTDNEP1 interacts directly with Eps8L2, a relationship critical for nuclear positioning and cell migration. This interaction contributes to the formation and regulation of dorsal actin cables necessary for TAN line engagement during nuclear movement^56^. Furthermore, CTDNEP1 has been identified as a novel factor in the negative regulation of osteoclastogenesis, functioning to prevent excessive formation of osteoclasts by downregulating RANKL signaling and Nfatc1 protein levels^57^.

Importantly, CTDNEP1, functioning as a phosphatase, has been recognized as a critical regulator within the MYC oncogenic pathway. This enzyme specifically targets and dephosphorylates serine 62 on the MYC protein, a modification that is essential for modulating MYC stability^25^. The phosphorylation at serine 62 is directly linked to the stabilization and activation of MYC, making its dephosphorylation by CTDNEP1 a key regulatory mechanism. In this study, we revealed that exosomal miR-1910-5p suppressed CTDNEP1 expression to activate MYC and its downstream targets, thereby promoting the colonization of BLCA cells in the lung. Taken together, we concluded that miR-1910-5p/CTDNEP1/MYC axis forms a signaling bridge between lung fibroblasts and BLCA cells, modulating the metastatic potential of BLCA cells to the lung. Given that CTDNEP1 remains an insufficiently characterized gene with complex and multifaceted functions, further research is warranted to elucidate its precise role in various cancer types. Such investigation could potentially uncover novel therapeutic targets. Notably, clinical correlation analyses revealed consistent trends in patient specimens that were directionally concordant with our mechanistic findings, although statistical significance was limited by the small number of available matched lung metastatic samples—a common challenge in studies of organ-specific metastatic niches. Such sample size constraints reduce statistical power and may obscure true biological associations in heterogeneous human tissues. Future studies leveraging larger, multi-center cohorts, together with high-resolution approaches such as digital pathology-based quantification, spatial transcriptomics and multiplexed RNA–protein co-detection, will be essential to further validate the clinical and biological relevance of this regulatory axis in BLCA lung metastasis.

In this study, we focused on the pivotal role of CAFs in shaping the lung metastatic niche of BLCA. CAFs are increasingly recognized as central architects of the metastatic niche through their coordinated regulation of extracellular matrix remodeling, metabolic adaptation and immune modulation^34,35^. Consistent with this concept, WNT7A-induced CAFs activation in our study was characterized by robust upregulation of α-SMA and FAP, accompanied by enhanced collagen gel contraction, indicating increased contractile activity and matrix remodeling capacity. These features are hallmarks of CAFs-mediated reorganization of the mechanical and structural properties of the metastatic microenvironment, which is known to facilitate tumor cell retention and colonization. Although the metabolic and immune regulatory functions of CAFs were not directly interrogated in this study, our snRNA-seq data revealed a pronounced enrichment of CAFs within BLCA lung metastatic lesions. This observation is consistent with previous reports demonstrating that activated CAFs support metastatic tumor cells through metabolic coupling and immune niche shaping. Moreover, sustained MYC activation in tumor cells downstream of CAFs-derived exosomal miR-1910-5p may further contribute to metabolic rewiring during metastatic colonization. Collectively, these findings suggest that WNT7A-driven CAFs activation orchestrates multiple dimensions of lung metastatic niche remodeling, extending beyond the regulation of tumor stemness. Future studies integrating spatial, metabolic and immunological profiling will be instrumental in fully elucidating the multifaceted roles of CAFs in metastatic niche formation.

To mechanistically interrogate metastatic colonization, we intentionally employed a tail vein injection model, which is widely regarded as a suitable approach to study the most rate-limiting and microenvironment-dependent stages of metastasis. It should be acknowledged, however, that this experimental model primarily captures the late stages of metastasis, particularly tumor cell survival, colonization and outgrowth within the pulmonary microenvironment, while bypassing earlier events such as primary tumor invasion, intravasation and systemic dissemination. Accordingly, our in vivo findings mainly reflect the role of tumor-derived WNT7A in remodeling the pulmonary metastatic niche and promoting metastatic colonization through CAFs activation and exosome-mediated signaling. Future studies employing orthotopic implantation or spontaneous metastasis models will be valuable to further determine whether WNT7A–CAFs crosstalk also contributes to earlier steps of the metastatic cascade.

Taken together, our results reveal the existence of a reciprocal activation loop between BLCA cells and lung fibroblasts during the process of BLCA lung metastasis. Tumor-derived WNT7A induces the transformation of lung NFs into CAFs, thereby remodeling the metastatic niche in the lung. CAFs, through exosomal delivery of miR-1910-5p, feed back to BLCA cells colonizing the lung, promoting the activation of the MYC signaling pathway by inhibiting the expression of the CTDNEP1. This, in turn, enhances the malignant properties of the BLCA cells, facilitating their colonization and eventually leading to the formation of lung metastatic lesions (Fig. 10g).

## Supplementary information

Supplementary information
