# Supplementary material for: Tumor-derived WNT7A reprograms pulmonary fibroblasts to remodel the metastatic niche and promote bladder cancer lung metastasis
Source: Exp Mol Med. 2026 Jun 3;58(6):1806–23. doi: 10.1038/s12276-026-01735-x (PMC13323384; doi:10.1038/s12276-026-01735-x)
Supplement: Supplementary file 1 — Supplementary information [file 12276_2026_1735_MOESM1_ESM.pdf]

Supplementary information

Tumor-Derived WNT7A reprograms pulmonary fibroblasts to remodel the metastatic niche and promote bladder cancer lung metastasis

Zhengnan Huang[1,2,7], Yilin Yan[3,7], Xinan Wang[1,7], Huaxing Li[4], Jingming Zhuang[4], Xiangqian Cao[3], Yang Wang[3], Denglong Wu[1] ✉ and Bing Shen[1,3,4,5,6] ✉

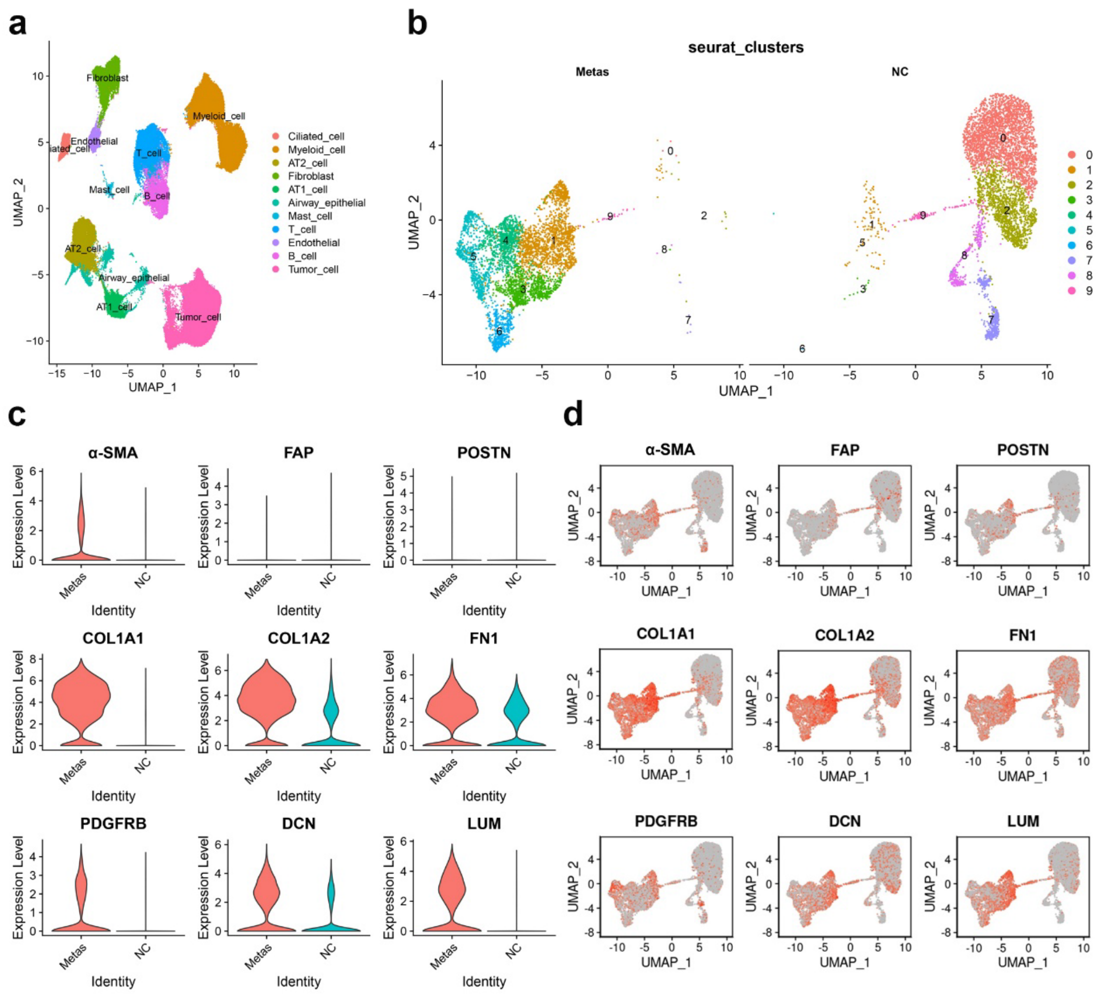

Supplementary Fig. 1 Characterization of fibroblast heterogeneity within the BLCA lung metastatic niche. a. Integrated UMAP visualization of snRNA-seq data from our BLCA lung metastasis samples (n=6) and normal control lung tissues (n=7) from GSE171524 dataset. **b.** Unsupervised subclustering of fibroblasts extracted from the integrated dataset identifies transcriptionally distinct subpopulations associated with metastatic (Meta) and normal control (NC) niches. **c.** Violin plots illustrating expression level of canonical CAFs markers across fibroblast subclusters. **d.** Feature plots visualizing spatial expression pattern of CAFs markers within the fibroblast UMAP space.

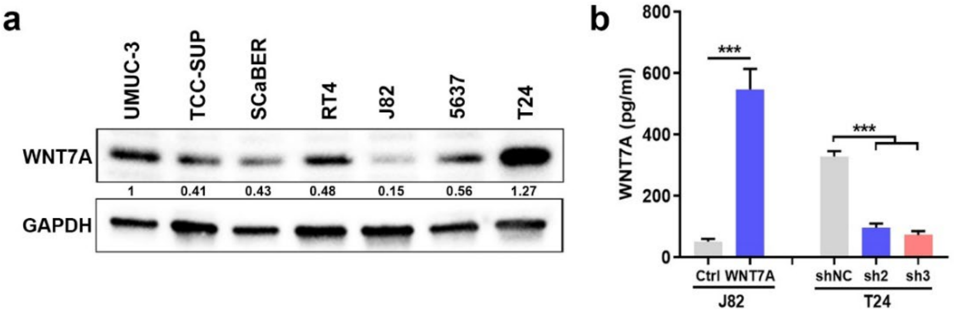

Supplementary Fig. 2 Expression analysis of WNT7A. a. Western blot analysis of endogenous WNT7A expression in multiple BLCA cell lines. **b.** ELISA analysis of WNT7A secretion level in WNT7A-overexpressing J82 cells or WNT7A-knockdown T24 cells. The statistical data are presented as mean ± SD, and the error bars represent the means of three independent experiments. ***$p < 0.001$.

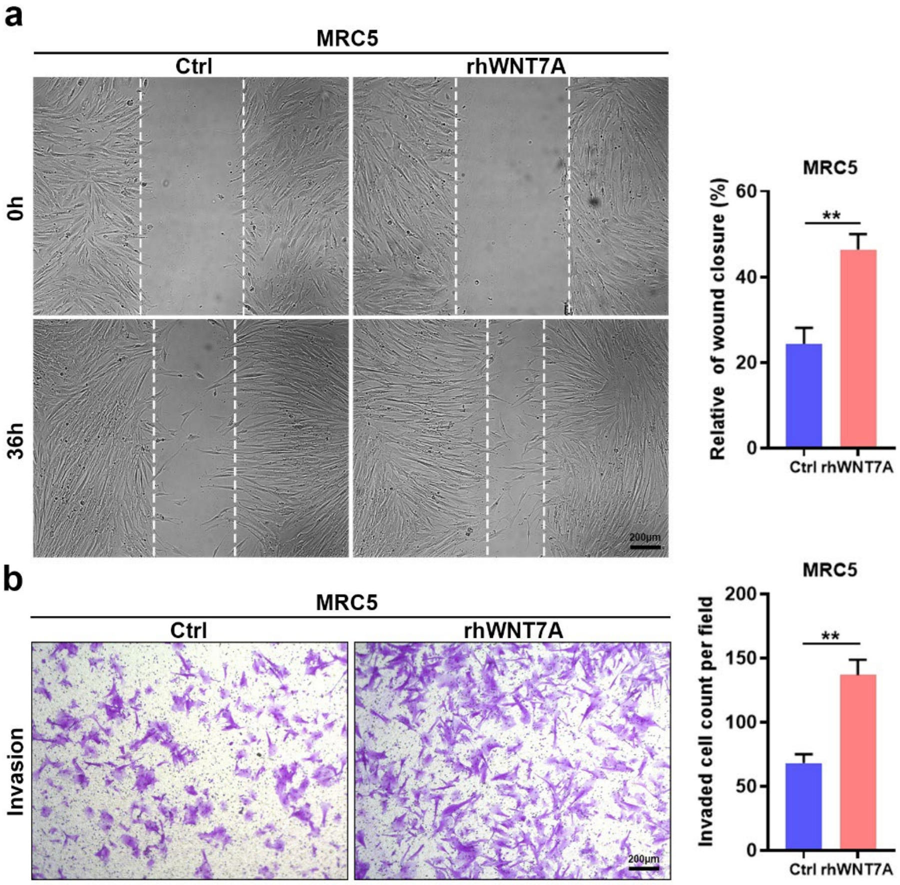

Supplementary Fig. 3 rhWNT7A promotes the migratory and invasive capacities of MRC5 fibroblasts. a. Wound healing assays of MRC5 after treated with rhWNT7A. Scale bar, 200 μm. **b.** Invasion assays of MRC5 after treated with rhWNT7A. Scale bar, 200 μm. The statistical data are presented as mean ± SD, and the error bars represent the means of three independent experiments. $**p < 0.01$.

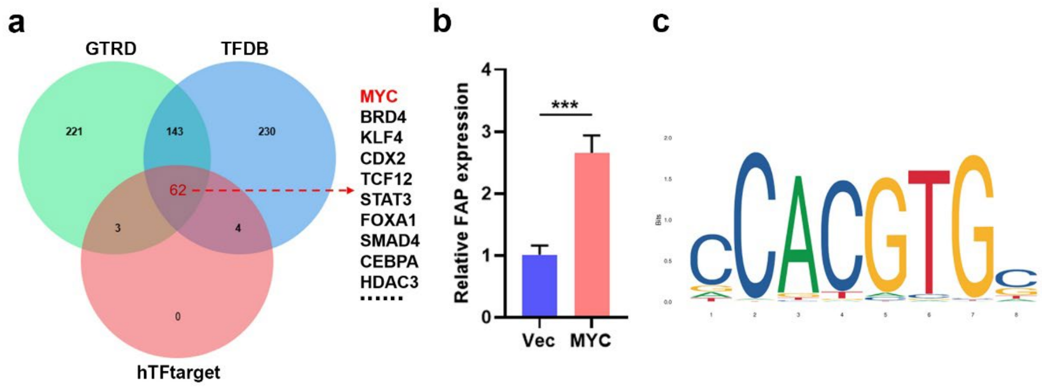

Supplementary Fig. 4 MYC is a potential transcriptional regulator of FAP. a. Venn diagram of the FAP putative transcription factors predicted by hTFtarget, TFDB, and GTRD. **b.** qPCR analysis of FAP expression in MYC-overexpressing MRC5 cells. **c.** Predicted sequences of MYC motif in FAP promoter by JASPAR.

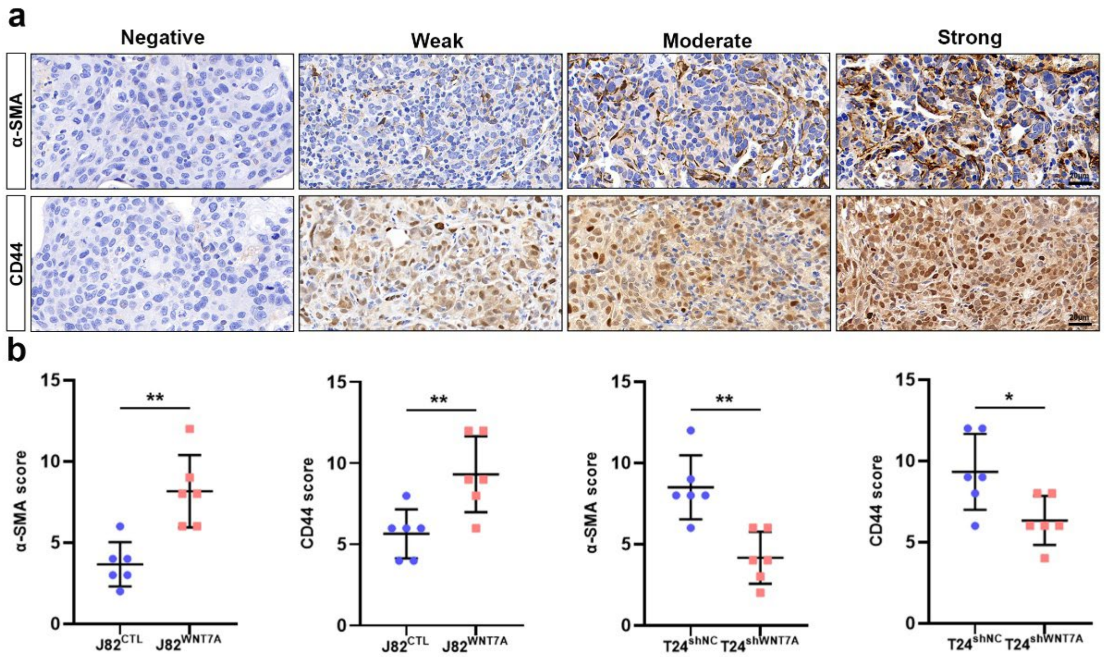

Supplementary Fig. 5 Analysis and quantification of α-SMA and CD44 expression in lung metastases. a. Representative images of varying staining intensities of α-SMA and CD44 in the metastatic foci from BALB/c nude mice. Scale bar, 20 μm. **b.** Quantification of immunohistochemical staining scores for α-SMA and CD44 across the experimental groups. The statistical data are presented as mean ± SD, and the error bars represent the means of six independent biological mouse samples. $*p < 0.05$, $**p < 0.01$.

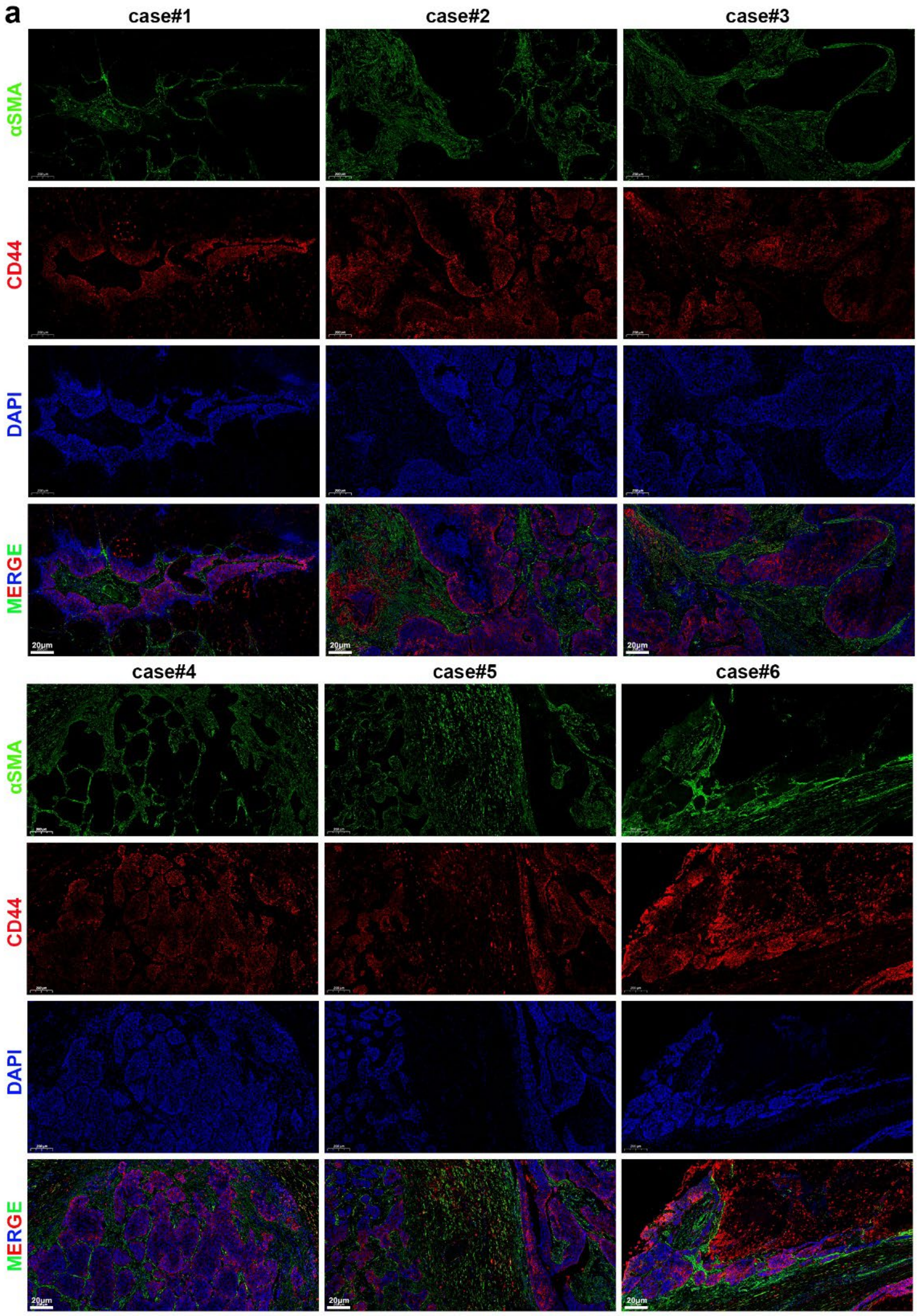

Supplementary Fig. 6 Characteristics of the pulmonary metastatic microenvironment in patients with BLCA lung metastasis. **a**. Immunofluorescence analysis of α-SMA and CD44 expression in patient specimens with BLCA lung metastasis. Scale bars, 20 μm.

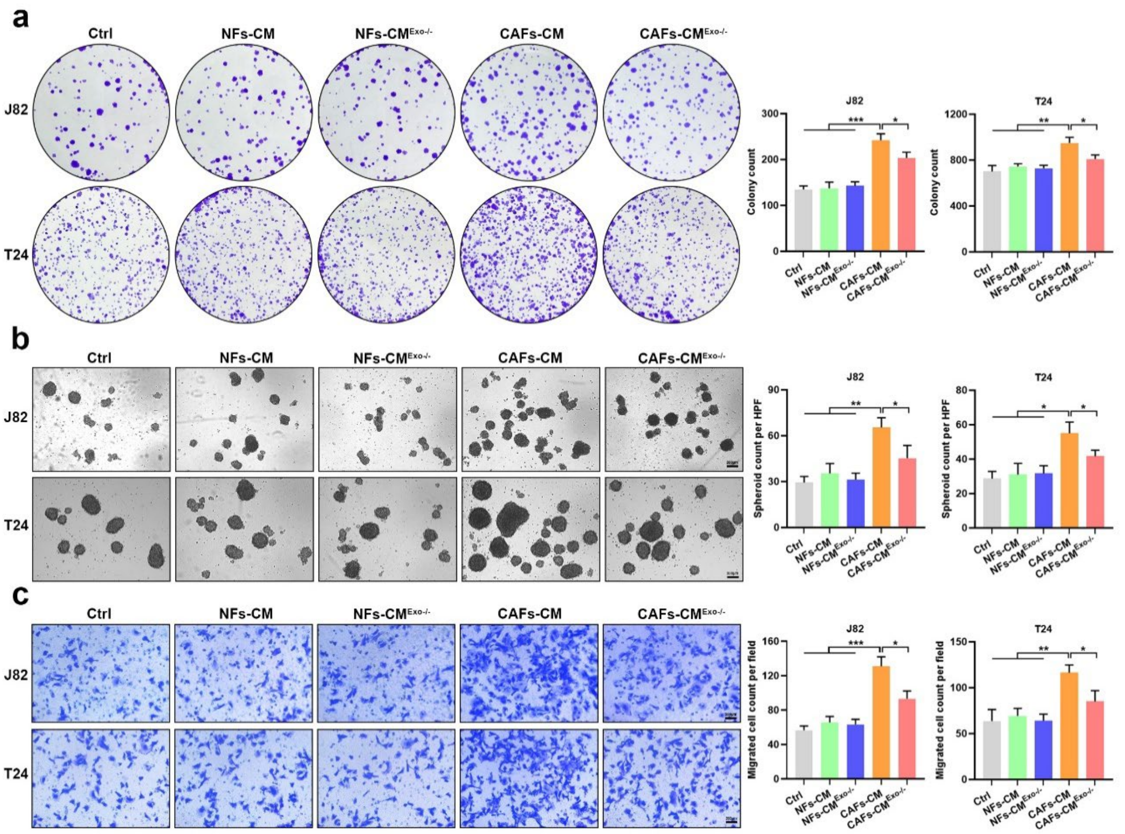

Supplementary Fig. 7 Effects of exosome depletion in NFs/CAFs-CM on BLCA cell proliferation, sphere formation, and migration. a. The impact of CM from NFs and CAFs, with or without the exosome depletion via ultracentrifugation, on the proliferative capacity of BLCA cells. **b.** The impact of CM from NFs and CAFs, with or without the exosome depletion via ultracentrifugation, on the sphere-forming ability of BLCA cells. Scale bars, 200 μm. **c.** The impact of CM from NFs and CAFs, with or without the exosome depletion via ultracentrifugation, on the migratory ability of BLCA cells. Scale bars, 200 μm. The statistical data are presented as mean ± SD, and the error bars represent the means of three independent experiments. *$p < 0.05$, **$p < 0.01$, ***$p < 0.001$.

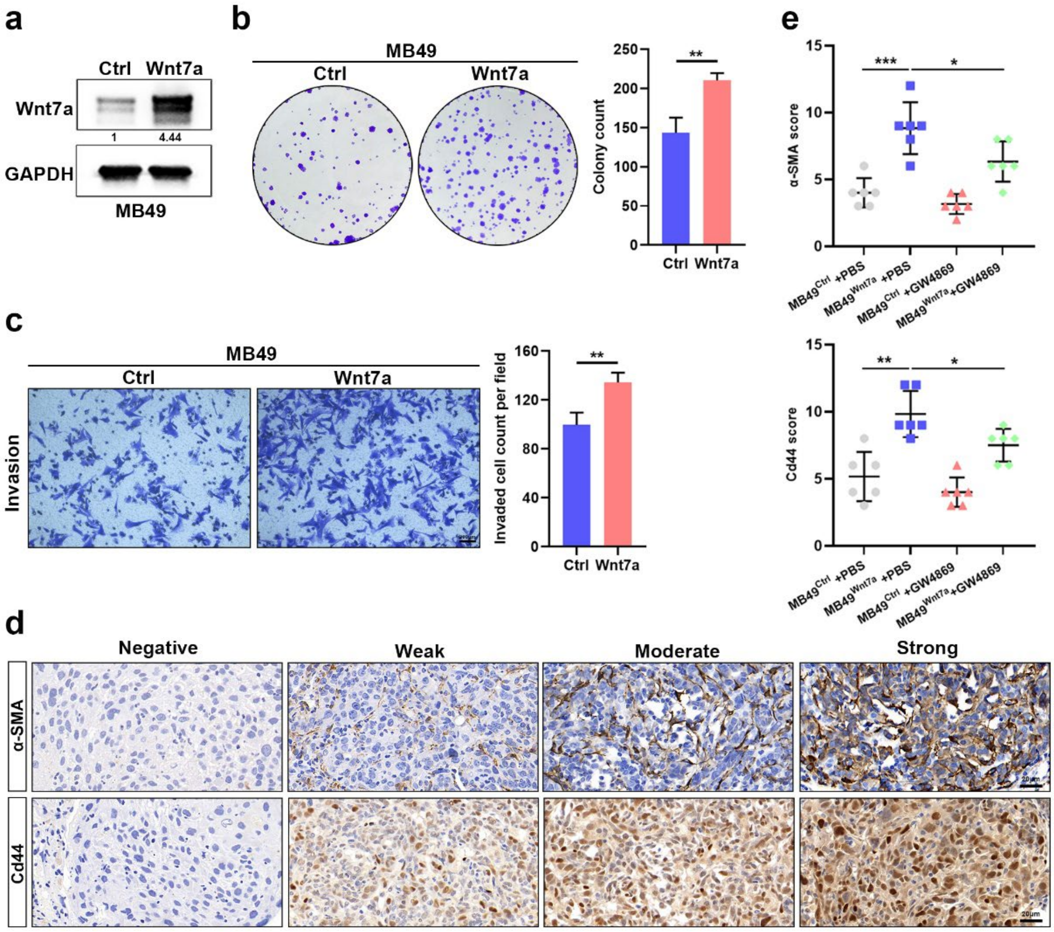

Supplementary Fig. 8 Wnt7a enhances the proliferative and migratory capacities of MB49 cells. a. Western blot analysis of confirmed overexpression of Wnt7a in MB49 cells. **b, c.** The impact of Wnt7a overexpression on the proliferative and migratory capacity of MB49 cells. Scale bar, 200 μm. **d.** Representative images of varying staining intensities of α-SMA and Cd44 in the metastatic foci from C57BL/6 mice. Scale bar, 20 μm. **e.** Quantification of immunohistochemical staining scores for α-SMA and Cd44 across the experimental groups. The statistical data are presented as mean ± SD, and the error bars represent the means of three independent experiments. $*p < 0.05$, $**p < 0.01$, $***p < 0.001$.

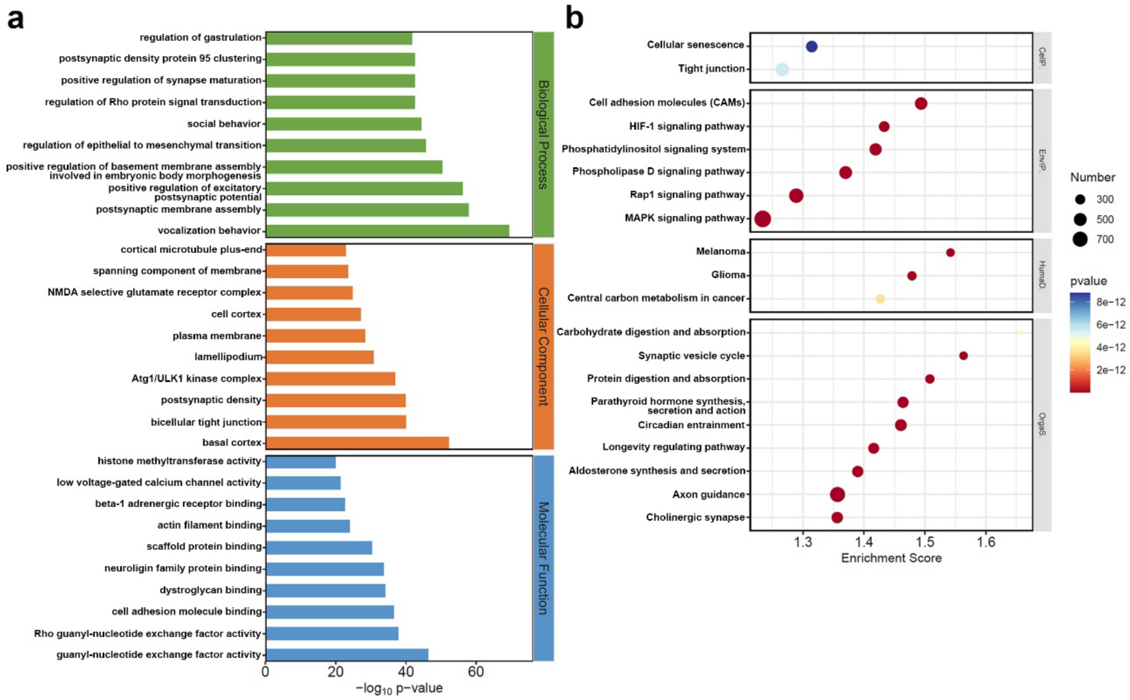

Supplementary Fig. 9 GO and KEGG analysis of differentially expressed miRNAs.

a. GO enrichment analysis of differentially expressed miRNAs. **b**. KEGG pathway enrichment analysis of differentially expressed miRNAs.

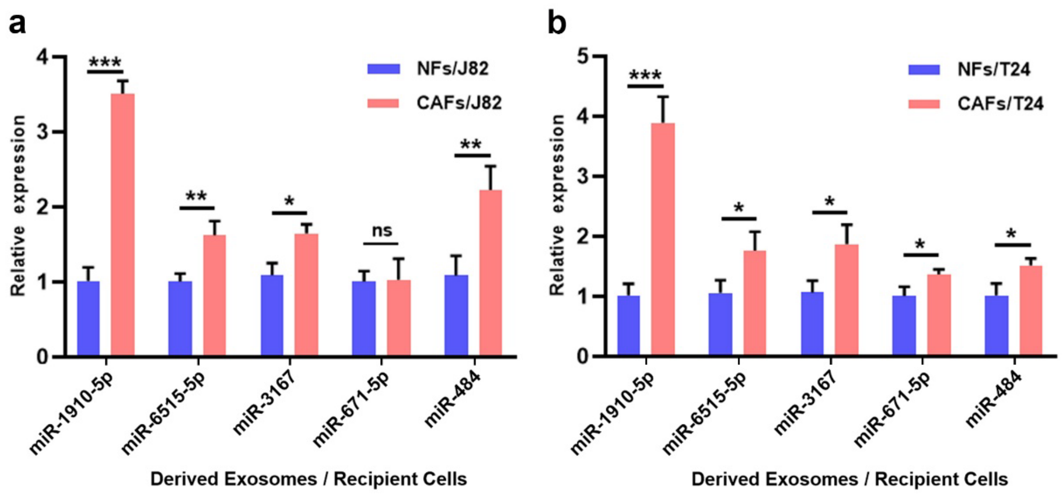

Supplementary Fig. 10 Identification and verification of differential miRNA expression. a, b. qPCR analysis of miR-1910-5p, miR-6515-5p, miR-3167, miR-671-5p and miR-484 expression in BLCA cells after incubating with exosomes from NFs or CAFs. The statistical data are presented as mean ± SD, and the error bars represent the means of three independent experiments. *$p < 0.05$, **$p < 0.01$, ***$p < 0.001$. ns nonsignificant.

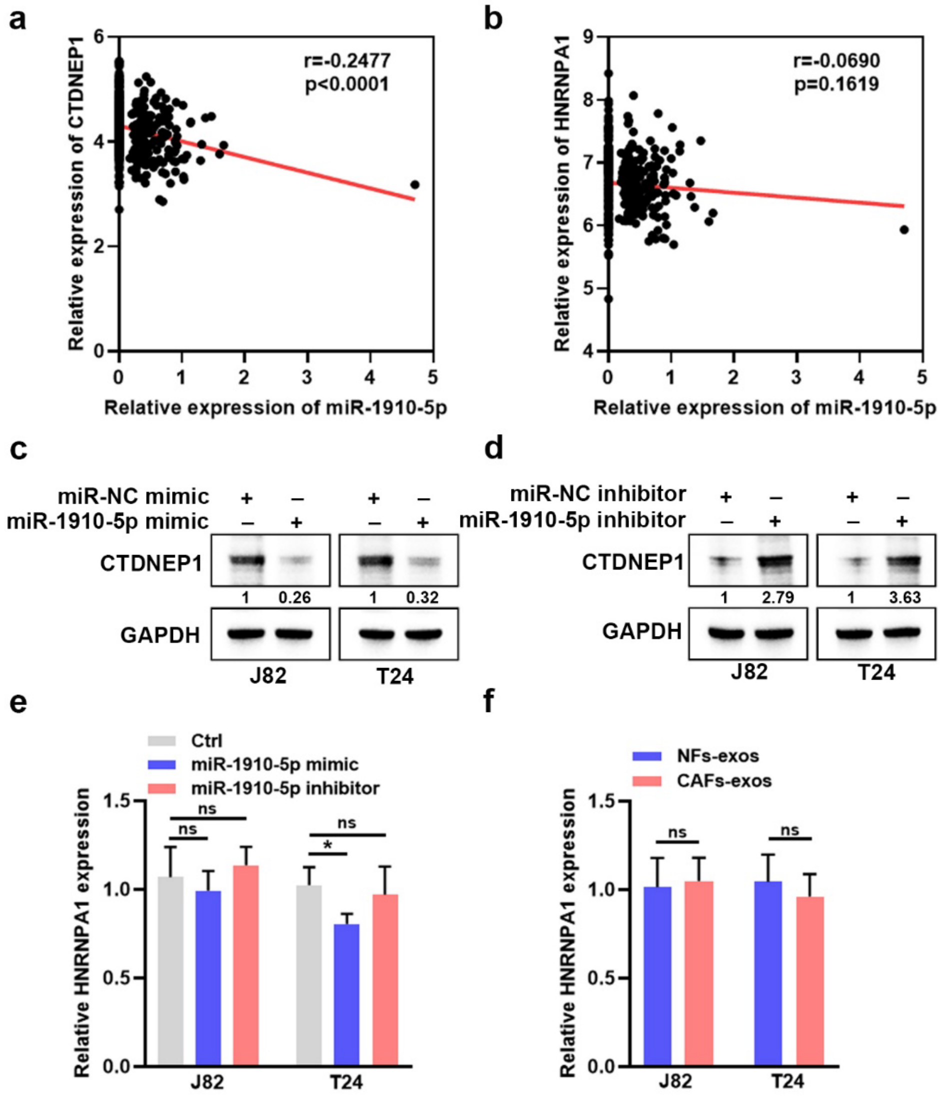

Supplementary Fig. 11 Analysis of the regulatory relationship between miR-1910-5p and CTDNEP1/HNRNPA1. a. The correction between CTDNEP1 and miR-1910-5p were determined from TCGA. **b.** The correction between HNRNPA1 and miR-1910-5p were determined from TCGA. **c**. Western blot analysis of CTDNEP1 expression in BLCA cells after treated with miR-1910-5p mimic. **d**. Western blot analysis of CTDNEP1 expression in BLCA cells after treated with miR-1910-5p inhibitor. **e.** qPCR analysis of HNRNPA1 expression in BLCA cells after treated with miR-1910-5p mimic or inhibitor. **f.** qPCR analysis of HNRNPA1 expression in BLCA cells after incubation with exosomes from NFs or CAFs. The statistical data are presented as mean ± SD, and the error bars represent the means of three independent experiments. *p < 0.05, ns nonsignificant.

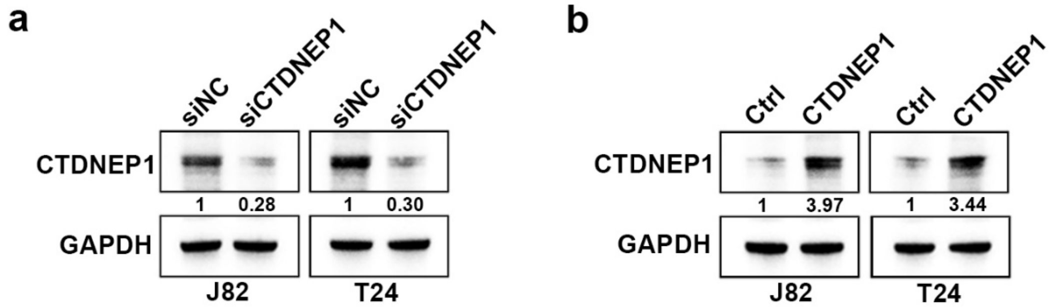

Supplementary Fig. 12 Validation of CTDNEP1 knockdown and overexpression. **a.** Western blot analysis of confirmed knockdown of CTDNEP1 in BLCA cells. **b.** Western blot analysis confirmed overexpression of CTDNEP1 in BLCA cells.

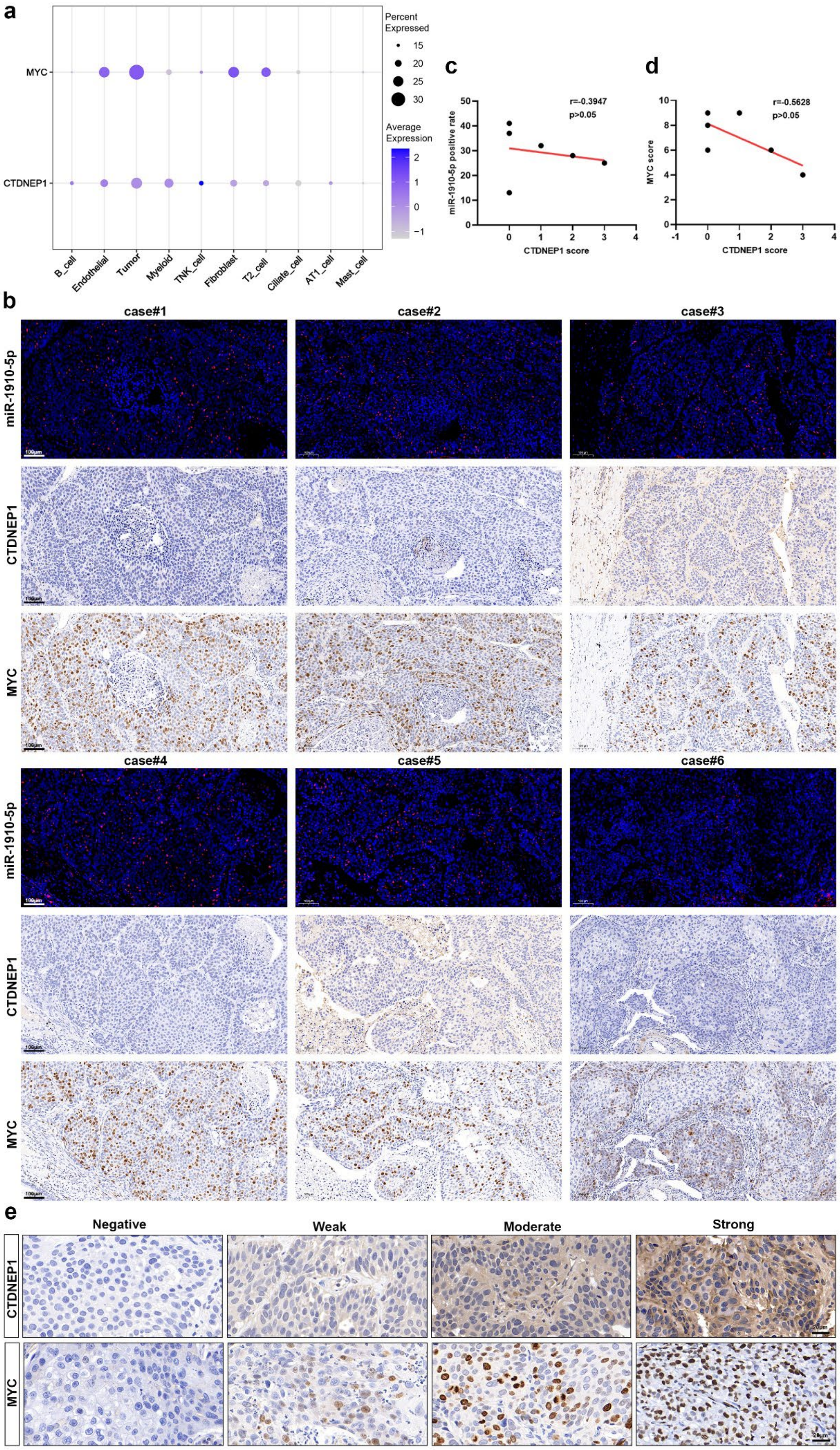

a
Percent Expressed
15
20
25
30
Average Expression
2
1
0
−1
MYC
CTDNEP1
B_cell
Endothelial
Tumor
Myeloid
TNK_cell
Fibroblast
T2_cell
Ciliate_cell
AT1_cell
Mast_cell
c
miR-1910-5p positive rate
CTDNEP1 score
r=-0.3947
p>0.05
d
MYC score
CTDNEP1 score
r=-0.5628
p>0.05
b
case#1
case#2
case#3
case#4
case#5
case#6
miR-1910-5p
CTDNEP1
MYC
e
Negative
Weak
Moderate
Strong
CTDNEP1
MYC

Supplementary Fig. 13 Clinical and histopathological validation of the WNT7A/miR-1910-5p/CTDNEP1/MYC axis in human BLCA lung metastases. a. Dot plot of MYC and CTDNEP1 expression across different cell types from the snRNA-seq analysis. **b.** FISH of miR-1910-5p in combination with IHC staining of CTDNEP1 and MYC on human BLCA lung metastatic tissue sections. Scale bars, 100 μm. **c.** Correlation analysis between miR-1910-5p FISH signal intensity and CTDNEP1 IHC score in human BLCA lung metastatic tissues. **d.** Correlation analysis between CTDNEP1 and MYC expression, as quantified by IHC scoring, in human BLCA lung metastatic tissues. **e.** Representative images of varying staining intensities of CTDNEP1 and MYC in the human BLCA lung metastatic tissue. Scale bar, 20 μm.

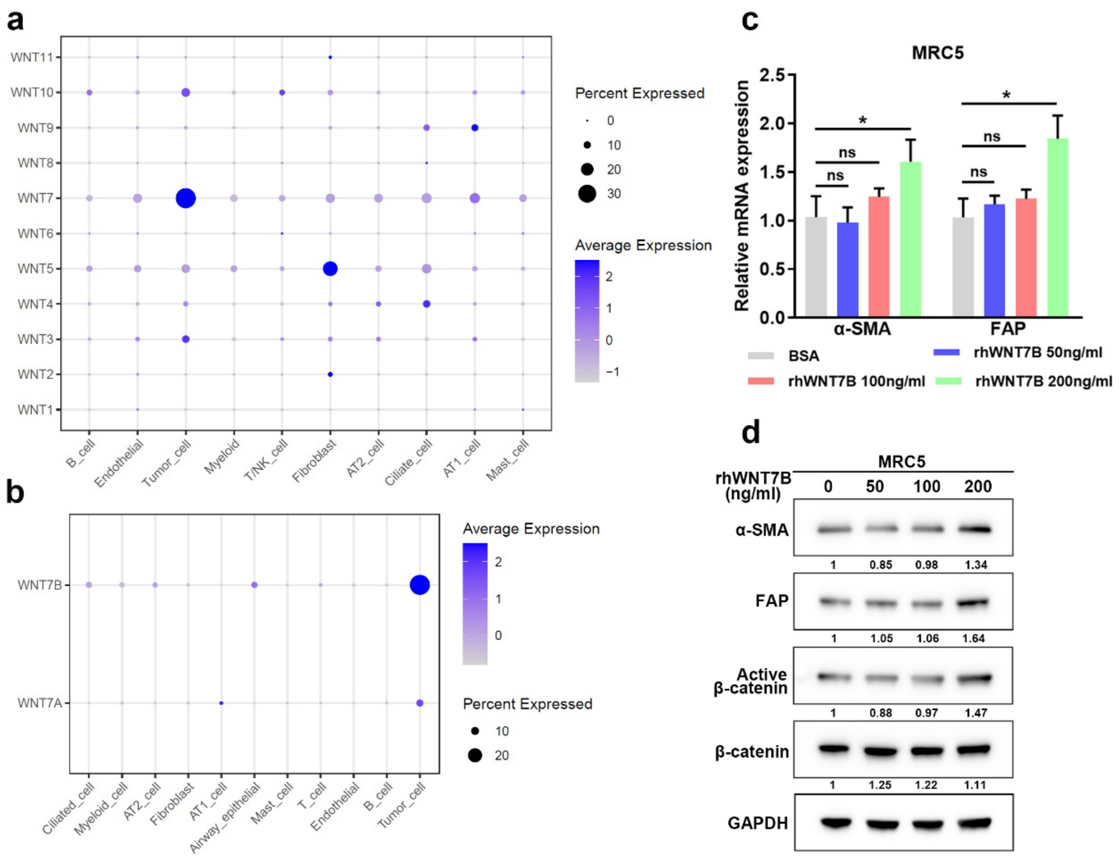

Supplementary Fig. 14 Investigation of the effects of WNT7B on lung fibroblast activation. a. Dot plot of Wnt gene family expression profiles across different cell types from the snRNA-seq analysis. **b.** Dot plot of WNT7A and WNT7B expression across different cell types from the snRNA-seq analysis. **c.** qPCR analysis of α-SMA and FAP expression in MRC5 after stimulation with rhWNT7B. **d.** Western blot analysis of specific proteins in MRC5 after stimulation with rhWNT7B. The statistical data are presented as mean ± SD, and the error bars represent the means of three independent experiments. *$p < 0.05$, ns nonsignificant.

Supplementary Table 1 Sequences of primers, shRNAs, siRNAs, miRNA mimic, and inhibitor

**Primers for qPCR**		**Sequences**
β-actin	Forward	5'-CATGTACGTTGCTATCCAGGC-3'
	Reverse	5'-CTCCTTAATGTCACGCACGAT-3'
WNT7A	Forward	5'-CTGTGGCTGCGACAAAGAGAA-3'
	Reverse	5'-GCCGTGGCACTTACATTCC-3'
CTDNEP1	Forward	5'-GACACTTATTCACTCCCACC-3'
	Reverse	5'-CCACAGCAGAGCCATAGA-3'
α-SMA	Forward	5'-GTGTTGCCCCTGAAGAGCAT-3'
	Reverse	5'-GCTGGGACATTGAAAGTCTCA-3'
FAP	Forward	5'-CAAAGGCTGGAGCTAAGAATCC-3'
	Reverse	5'-ACTGCAAACATACTCGTTCATCA-3'
KHSRP	Forward	5'-GCGGGGAGACCATTAAGCAG-3'
	Reverse	5'-CCGAGATCCGTACTCATTCCG-3'
ACO1	Forward	5'-CGCAGCACAAGAACATAGAAGT-3'
	Reverse	5'-CATTGCAGCAAAGTCAACCAC-3'
RBMX	Forward	5'-TCACCCCTCCGACTCACC-3'
	Reverse	5'-TTGGTGGCTTGTTCCACCTT-3'
MYC	Forward	5′-GTCAAGAGGCGAACACACAAC-3′
	Reverse	5′-TTGGACGGACAGGATGTATGC -3′
hsa-miR-1910-5p	Forward	5'-CGCCAGTCCTGTGCCTG-3'
hsa-miR-6515-5p	Forward	5'-GCGTTGGAGGGTGTGGAA-3'
hsa-miR-3167	Forward	5'-GCGCGAGGATTTCAGAAATAC-3'
hsa-miR-10401-3p	Forward	5'-AACCTCGCCGTCCCGC-3'
hsa-miR-671-5p	Forward	5'-AGGAAGCCCTGGAGGGG-3'
hsa-miR-3129-5p	Forward	5'-GCGCGGCAGTAGTGTAGAGAT-3'
hsa-miR-3190-3p	Forward	5'-CGTGTGGAAGGTAGACGGC-3'
hsa-miR-2110	Forward	5'-TTGGGGAAACGGCCGC-3'
hsa-miR-629-5p	Forward	5'-GCGTGGGTTTACGTTGGG-3'
hsa-miR-484	Forward	5'-GCGTCAGGCTCAGTCCCCT-3'
cel-miR-39	Forward	5'-GGTCACCGGGTGTAAATCAGCTTG-3'
Human U6 snRNA	Forward	5'-CTCGCTTCGGCAGCACA-3'
**Primers for ChIP analysis**		**Sequences**
α-SMA promoter	Forward	5'-CTGAGAGGACTAGGGGAAGAGG-3'
	Reverse	5'-CTGAGTGAAGGCTGAGGAGG-3'
FAP promoter binding site 1	Forward	5'-GCATATGGAGATACCTACAAG-3'
	Reverse	5'-TCTGAGGGCCAAGAACTGT-3'
FAP promoter binding site 2	Forward	5'-TCCCGTGGAGATACCTACAAG-3'
	Reverse	5'-TCTGAGGGCCAAGAACTGT-3'
FAP promoter binding site 3	Forward	5'-TCACTTGGAGATACCTACAAG-3'
	Reverse	5'-TTCATTTGAGGGCCAAGAA-3'

**siRNA**	**Sequences**
siRNA-NC	5′-GAACGUCCCAUGAUGUGAATT-3′
siRNA-CTDNEP1	5’-GUACCAAACUGUUCGAUAUTT-3’
siRNA-KHSRP-1	5’-CCCGAGAAGAUUGCUCAUAUATT-3’
siRNA-KHSRP-2	5’-GACUUCAAUGACAGAAGAGUATT-3’
siRNA-ACO1-1	5’-CCAGGAAAGAAAUUCUUCAAUTT-3’
siRNA-ACO2-2	5’-CCAGGAAAGAAAUUCUUCAAUTT-3’
siRNA-RBMX-1	5’-CAUCAAGAGGAUAUAGCGAUATT-3’
siRNA-RBMX-2	5’-CUUGAAGCAGUAUUUGGCAAATT-3’
**shRNA**	**Sequences**
sh-NC	5′-CAACAAGATGAAGAGCACCAA-3′
shWNT7A -1	5′-GCGCAAGCATCATCTGTAACA-3′
shWNT7A -2	5′-GCCATCATCGTCATAGGAGAA-3′
shWNT7A -3	5′-GGAGAACATGAAGCTGGAATG-3′
**mimic and inhibitor**	**Sequences**
miR-NC	5’-CAGUACUUUUGUGUAGUACAA-3’
miR-1910-5p mimic	5’-CCAGUCCUGUGCCUGCCGCCU-3’
miR-1910-5p inhibitor	5’-AGGCGGCAGGCACAGGACUGG-3’

Supplementary Table 2 Primary antibodies used in this study

Antigens	Manufacturer	Application
α-SMA	Abcam	1:100 for IF; 1:1000 for WB; 1:200 for IHC
FAP	Abcam	1:100 for IF; 1:1000 for WB
WNT7A	Abcam	1:1000 for WB
GAPDH	Cell Signaling Technology	1:1000 for WB
Active β-catenin	Cell Signaling Technology	1:1000 for WB
β-catenin	Cell Signaling Technology	1:100 for IF; 1:1000 for WB; 1:50 for IP
LEF-1	Cell Signaling Technology	1:50 for IP
IgG	Proteintech	1:50 for IF
CD44	Proteintech	1:100 for IF; 1:500 for IHC
Calnexin	Abcam	1:1000 for WB
ALIX	Abcam	1:1000 for WB
CD63	Abcam	1:1000 for WB
CD9	Abcam	1:1000 for WB
CD81	Abcam	1:1000 for WB
KHSRP	Cell Signaling Technology	1:1000 for WB
ACO1	Abcam	1:1000 for WB
RBMX	Cell Signaling Technology	1:1000 for WB; 1:50 for IP
p-S62 MYC	Abcam	1:1000 for WB
MYC	Proteintech	1:5000 for WB; 1:50 for IP; 1:500 for IHC
CDK4	Proteintech	1:1000 for WB
SNAI1	Cell Signaling Technology	1:1000 for WB
SOX2	Proteintech	1:500 for WB
CTDNEP1	Invitrogen	1:500 for WB; 1:100 for IHC
H3 histone	Cell Signaling Technology	1:1000 for WB
Rabbit IgG Isotype Control	Proteintech	1:50 for IF
